# Prediction of pathological complete response after neoadjuvant chemotherapy for HER2-negative breast cancer patients with routine immunohistochemical markers

**DOI:** 10.1186/s13058-025-01960-8

**Published:** 2025-01-24

**Authors:** Lothar Häberle, Ramona Erber, Paul Gass, Alexander Hein, Melitta Niklos, Bernhard Volz, Carolin C. Hack, Rüdiger Schulz-Wendtland, Hanna Huebner, Chloë Goossens, Matthias Christgen, Thilo Dörk, Tjoung-Won Park-Simon, Andreas Schneeweiss, Michael Untch, Valentina Nekljudova, Sibylle Loibl, Arndt Hartmann, Matthias W. Beckmann, Peter A. Fasching

**Affiliations:** 1 Department of Gynecology and Obstetrics, Erlangen University Hospital, Comprehensive Cancer Center Erlangen-EMN, Friedrich Alexander University of Erlangen–Nuremberg, Erlangen, Germany; 2 Biostatistics Unit, Department of Gynecology and Obstetrics, Erlangen University Hospital, Erlangen, Germany; 3 Institute of Pathology, Erlangen University Hospital, Comprehensive Cancer Center Erlangen-EMN, Friedrich Alexander University of Erlangen–Nuremberg, Erlangen, Germany; 4 Ansbach University of Applied Sciences, Ansbach, Germany; 5 Institute of Diagnostic Radiology, Erlangen University Hospital, Comprehensive Cancer Center Erlangen-EMN, Friedrich Alexander University of Erlangen–Nuremberg, Erlangen, Germany; 6 Institute of Pathology, Hannover Medical School, Hannover, Germany; 7 Gynecology Research Unit, Hannover Medical School, Hannover, Germany; 8 Department of Gynecology and Obstetrics, Hannover Medical School, Hannover, Germany; 9 National Center for Tumor Diseases, University Hospital and German Cancer Research Center, Heidelberg, Germany; 10 Department of Gynecology and Obstetrics, Helios Clinic Berlin-Buch, Berlin, Germany; 11 German Breast Group, Neu-Isenburg, Germany

**Keywords:** pCR, Prediction model, Breast cancer, Neoadjuvant chemotherapy, Immunohistochemistry, Clinical data

## Abstract

**Background:**

Pathological complete response (pCR) is an established surrogate marker for prognosis in patients with breast cancer (BC) after neoadjuvant chemotherapy. Individualized pCR prediction based on clinical information available at biopsy, particularly immunohistochemical (IHC) markers, may help identify patients who could benefit from preoperative chemotherapy.

**Methods:**

Data from patients with HER2-negative BC who underwent neoadjuvant chemotherapy from 2002 to 2020 (n = 1166) were used to develop multivariable prediction models to estimate the probability of pCR (pCR-prob). The most precise model identified using cross-validation was implemented in an online calculator and a nomogram. Associations among pCR-prob, prognostic IHC3 distant recurrence and disease-free survival were studied using Cox regression and Kaplan–Meier analyses. The model’s utility was further evaluated in independent external validation cohorts.

**Results:**

273 patients (23.4%) achieved a pCR. The most precise model had across-validated area under the curve (AUC) of 0.84, sensitivity of 0.82, and specificity of 0.71. External validation yielded AUCs between 0.75 (95% CI, 0.70–0.81) and 0.83 (95% CI, 0.78–0.87). The higher the pCR-prob, the greater the prognostic impact of pCR status (presence/absence): hazard ratios decreased from 0.55 (95% central range, 0.07–1.77) at 0% to 0.20 (0.11–0.31) at 50% pCR-prob. Combining pCR-prob and IHC3 score further improved the precision of disease-free survival prognosis.

**Conclusions:**

A pCR prediction model for neoadjuvant therapy decision-making was established. Combining pCR and recurrence prediction allows identification of not only patients who benefit most from neoadjuvant chemotherapy, but also patients with a very unfavorable prognosis for whom alternative treatment strategies should be considered.

**Supplementary Information:**

The online version contains supplementary material available at 10.1186/s13058-025-01960-8.

## Background

Pathological complete response (pCR) after neoadjuvant chemotherapy is a surrogate marker for prognosis in patients with early breast cancer (BC) [1, 2]. Particularly in patients with triple-negative or HER2-positive breast cancer, a pCR is strongly associated with a favorable prognosis [1, 2]. Some hormone receptor–positive, HER2-negative patients have an excellent prognosis despite low pCR rates [1, 2]. Ideally, biomarkers can help identify patients who have a good response to chemotherapy and serve to justify such treatment, especially when the presence or absence of a pCR has a substantial impact on survival. A survey among physicians confirmed the general interest in a prediction tool for pCR[3]. The present study focuses on HER2-negative disease because, for HER2-positive patients, the indication for chemotherapy is usually established independently of biomarker values: most of these patients have been shown to benefit from combined chemotherapy with trastuzumab[4].

Although many molecular biomarkers have been shown to be associated with pCR, and some mRNA-based multigene assays have been used to predict the response to chemotherapy[5], the full potential of immunohistochemistry (IHC) may not yet have been fully explored.

IHC markers, including estrogen receptor (ER), progesterone receptor (PgR), HER2, and in some institutions Ki-67 as well, are often used for clinical decision making. Although ER, PgR, and Ki-67 assessments are usually reported as the percentage of positively stained cells, dichotomized information (positive vs negative) is mainly used in clinical trials and scientific analyses. For routine clinical use, some cutoff values have been discussed, but these cut-off points have changed in some cases. The cutoff points for ER/PgR, for example, have decreased from 10% to 1% over time [6–10]. These cutoff values were chosen in relation to responsiveness to antihormonal therapy but have nevertheless also been used to predict the efficacy of chemotherapy [1, 2, 11–14].

Combined information allows individualized prediction of pCR that goes beyond the single dichotomized biomarker approach currently used. The primary objective of this study was to develop various prediction tools to estimate a patient’s likelihood of pCR on the basis of clinical predictors and the IHC biomarkers ER, PgR, and Ki-67—either as assessed during routine clinical work or categorically using established or newly identified thresholds. The most precise tool is described in detail and has been validated in several independent external samples.

## Methods

### Primary study population

**Fig. 1:**
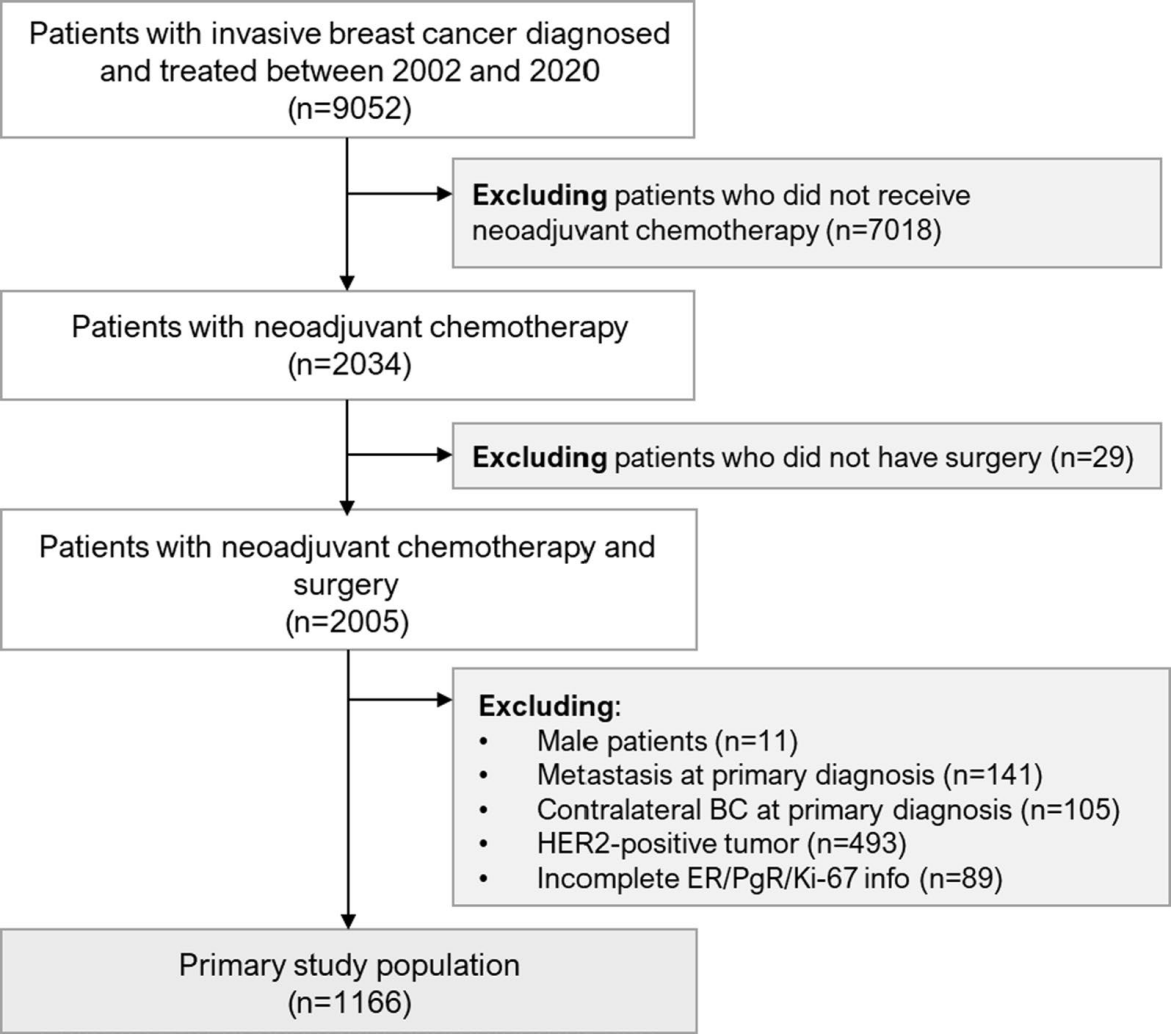
Patient flow diagram for the primary study population (CONSORT diagram). BC, breast cancer; ER, estrogen receptor (expression); PgR, progesterone receptor (expression)

This retrospective, single-center, hospital-based observational study included 1166 patients with HER2-negative BC from the Erlangen Neoadjuvant Study Breast (ERNEST-B) study [12] who underwent neoadjuvant chemotherapy from 2002 to 2020, were ≥ 18 years of age, and had available information about their pCR status. Patients with metastases or contralateral breast cancer at primary diagnosis and patients with incomplete biomarker information were excluded. Approval for the analyses was obtained from the ethics committee of the University of Erlangen–Nuremberg. Further information is provided in Fig. 1 and the Supplement.

Data were collected prospectively in accordance with the breast center certification requirements of the German Cancer Society. Follow-up data were collected for ≤ 10 years after the primary diagnosis.

All histopathological parameters were documented from the original pathology reports. Tumor grade, tumor type, HER2 status, ER/PgR expression, and Ki-67 staining were assessed as part of clinical routine testing on formalin-fixed, paraffin-embedded tumor tissue at initial diagnosis. The staining procedures are described in the Supplement.

### Study outcome

pCR was defined as a complete absence of tumor cells from the breast (ypT0) and lymph nodes (ypN0) after chemotherapy at the time of surgery.

Disease-free survival (DFS) was defined as the period from the date of diagnosis to either the earliest date of disease progression (ie, distant metastasis, local recurrence, or death from any cause) or to the last date the patient was known to be disease free within the 10-year maximum observation time.

### Univariable statistical analyses

The possibly nonlinear relationship between the biomarkers ER, PgR, and Ki-67 (each continuous, 0% to 100% of positively stained cells) and pCR (yes vs no) was described using natural cubic spline functions. Optimal cutoff values for these biomarkers were calculated using the minimum *p* value approach. Full details are provided in the Supplement.

### Developing pCR prediction models

Several logistic regression models were fitted to assess different usages of the biomarkers ER, PgR, and Ki-67 for predicting a patient’s likelihood of pCR. A logistic regression model (hereafter referred to as the basic model) was set up with established predictors for pCR: age at diagnosis (continuous), inverse body mass index (1/BMI, continuous), tumor stage (ordinal, cT1–cT4), grade (ordinal, grade 1–grade 3), lymph node status (categorical, cN0 vs cN +) and tumor type (categorical; ductal, lubular, other). Continuous predictors (age, 1/BMI) were used as cubic spline functions in which the degrees of freedom (*df;* 1 or 2) were determined using the Akaike information criterion (AIC). Missing predictor values were substituted by predicted expected values based on nonmissing data. All other prediction models were extensions of the basic model, including the biomarkers of interest.

Two logistic regression models were fitted with the biomarkers of interest as binary categorical predictors, one using established cutoff points (1% for ER and PgR [9], 14% for Ki-67 [15]) and the other using optimal cutoff points from the univariable analyses mentioned in the *Univariable Statistical Analyses* subsection. The biomarkers ER, PgR and Ki-67 were used continuously (0% to 100% of positively stained cells) as natural cubic spline functions, with 1 to 3 *df* in 27 (ie, 3^3^) further regression models considering all combinations of *df*. To improve prediction, shrinkage of regression coefficients after estimation was applied [16].

### Internal assessment of prediction models

The performance of the models in relation to calibration and discrimination was assessed using the mean squared error (MSE), receiver operating characteristic (ROC) curve, and area under the ROC curve (AUC). These measures were obtained by threefold cross-validation with 100 repetitions in order to obtain stable and realistic results [17–19]. In particular, all model-building steps were performed using training data, and the performance of the model was assessed using validation data that had not been used for model building. Apparent measures on the complete dataset were calculated to assess overfitting. In addition, the AIC was used as an alternative model performance measure. It was applied to the complete dataset and took overfitting into account by penalizing complex models.

The model with the smallest cross-validated MSE was considered the final model*,* which had the best usage of the biomarkers ER, PgR, and Ki-67 in comparison with the other usages [17, 18]. It was fitted on the complete dataset and analyzed in greater detail. An explicit formula for a patient’s predicted pCR probability (pCR-prob) was derived, implemented in an online calculator, and graphically presented as a nomogram. Model performance was also assessed using the Hosmer–Lemeshow calibration plot and χ^2^ test, as previously done [20]. Cross-validated sensitivities and specificities are presented. Spearman correlation coefficient ρ was calculated for pCR-prob and the IHC3 + C score, which incorporates ER, PgR, and Ki-67 (IHC3) and clinical predictors (C) and provides prognostic information on the risk of distant recurrence [21]. The IHC3 score is virtually identical to IHC4 when HER2 is negative [21].

The association between pCR-prob and the impact of pCR on the prognosis was analyzed using a Cox regression model with the following predictors: observed pCR status (yes vs no), pCR-prob obtained from the final logistic regression model as a cubic spline function with 2 *df,* and the interaction between the two predictors. To obtain hazard ratios for pCR (yes vs no) as a continuous function of pCR-prob, 20,000 random sample splittings were carried out in which the final model was fitted on half the data first and the Cox regression analysis was then performed on the remaining half.

Kaplan–Meier curves for DFS are shown in accordance with recently suggested pCR probability groups [3] and IHC3 + C risk classes.

### External validation of the final prediction model

The observational Hannover Breast Cancer Study (HaBCS [22], n = 338, see Supplement) and two randomized clinical trials (GeparSepto [23], n = 781; GeparOcto [24], n = 269; see Supplement) were each used to validate the final prediction model that had been fitted on the primary study dataset. The discrimination ability was assessed using the AUC. Its 95% CI was estimated using 10,000 bootstrap samples. Calibration was checked using a calibration plot in addition to a simple logistic regression model with observed pCR as the outcome and the logit of pCR-prob as the only predictor (hereafter termed the calibration model). If the intercept significantly (*p* < 0.05) differed from 0, or if the slope significantly differed from 1, then the calibration intercept and slope were used to update the original prediction model for future application in the validation cohort [19, 25].

The final prediction model was further assessed (AUC) in subgroups according to hormone receptor status to control for heterogeneity within and across study populations.

Calculations were carried out using the R system for statistical computing (version 4.1.1; R Foundation for Statistical Computing, Vienna, Austria).

## RESULTS

### Patients

In the primary study cohort, 273 (23.4%) of the 1166 patients achieved a pCR. In the validation cohorts, pCR rates ranged from 17.1% to 32.7% (Supplementary Table S1). The patient characteristics grouped by pCR status are shown in Table 1, and biomarker distributions in the primary cohort are shown in Supplementary Fig. S1.

**Table 1:** Characteristics of patients and tumors in the primary study cohort and validation cohorts relative to pCR status

Characteristic	Primary study population^a^ (n = 1166)		HaBCS^b^ (n = 338)		GeparSepto^b^ paclitaxel (n = 392)		GeparSepto^b^ nab-pacitaxel (n = 389)		GeparOcto^b^ ETC (n = 269)	
	No pCR (n = 893)	pCR (n = 273)	No pCR (n = 247)	pCR (n = 91)	No pCR (n = 325)	pCR (n = 67)	No pCR (n = 280)	pCR (n = 109)	No pCR (n = 181)	pCR (n = 88)
Age (years)	52.9 (11.5)	50.3 (12.4)	54.9 (12.4)	50.5 (11.2)	49.2 (11.0)	48.5 (11.2)	49.7 (10.2)	50.3 (9.5)	49.2 (10.8)	47.0 (10.2)
BMI (kg/m^2^)	26.4 (5.2)	25.1 (4.9)	25.1 (2.3)	25.4 (2.6)	26.5 (5.5)	25.9 (5.6)	26.6 (5.5)	26.0 (5.2)	26.2 (5.1)	26.6 (5.5)
*Tumor stage*										
cT1	185 (20.7)	110 (40.3)	36 (14.6)	23 (25.3)	96 (29.2)	31 (46.3)	79 (28.2)	52 (47.7)	64 (35.4)	36 (40.9)
cT2	567 (63.5)	148 (54.2)	179 (72.5)	62 (68.1)	177 (54.5)	34 (50.7)	158 (56.4)	50 (54.9)	90 (49.7)	48 (43.5)
cT3	48 (5.4)	5 (1.8)	15 (6.1)	4 (4.4)	32 (9.8)	1 (1.5)	24 (8.6)	2 (1.8)	20 (11.0)	3 (3.4)
cT4	93 (10.4)	10 (3.7)	17 (6.9)	2 (2.2)	20 (6.2)	1 (1.5)	19 (6.8)	5 (4.6)	7 (3.9)	1 (1.1)
*Grade*										
Grade 1	36 (4.0)	2 (0.7)	3 (1.2)	0 (0.0)	7 (2.2)	1 (1.5)	8 (2.9)	4 (3.7)	2 (1.1)	1 (1.1)
Grade 2	401 (44.9)	34 (12.5)	71 (28.7)	6 (6.6)	141 (43.4)	15 (22.4)	138 (49.3)	27 (24.8)	62 (34.3)	4 (4.5)
Grade 3	456 (51.1)	237 (86.8)	173 (70.0)	85 (93.4)	177 (54.5)	51 (76.1)	134 (47.9)	78 (71.6)	117 (64.6)	83 (94.3)
*Lymph node status*										
cN0	389 (43.6)	159 (58.2)	137 (55.5)	60 (65.9)	209 (64.3)	49 (73.1)	173 (61.8)	80 (73.4)	105 (58.0)	66 (75.0)
cN +	504 (56.4)	114 (41.8)	110 (44.5)	31 (34.1)	116 (35.7)	18 (26.9)	107 (38.2)	29 (26.6)	76 (32.0)	22 (25.0)
*Tumor type*										
Ductal	634 (71.0)	193 (70.7)	221 (89.5)	87 (95.6)	275 (84.6)	53 (79.1)	233 (83.2)	92 (84.4)	155 (85.6)	63 (71.6)
Lobular	94 (10.5)	5 (1.8)	17 (6.9)	1 (1.1)	18 (6.6)	2 (3.0)	22 (7.9)	1 (0.9)	0 (0.0)	0 (0.0)
Other	165 (18.5)	75 (27.5)	9 (3.6)	3 (3.3)	32 (9.4)	12 (17.9)	25 (8.9)	16 (14.7)	26 (14.4)	25 (28.4)
ER (continuous, 0%–100%)	55.7 (39.8)	12.9 (28.4)	55.5 (42.5)	9.7 (24.7)	52.4 (45.3)	18.5 (34.8)	60.8 (44.4)	15.6 (31.8)	28.8 (43.1)	3.0 (14.7)
*ER (categorical)*										
Negative (< 1%)	246 (27.5)	199 (72.9)	74 (30.0)	73 (80.2)	113 (34.8)	44 (65.7)	111 (39.6)	82 (75.2)	118 (65.2)	82 (93.2)
Positive (≥ 1%)	647 (72.5)	74 (27.1)	173 (70.0)	18 (19.8)	212 (65.2)	23 (34.3)	169 (60.4)	27 (24.8)	63 (34.8)	6 (6.8)
PgR (continuous, 0%–100%)	34.1 (36.4)	4.4 (14.8)	34.8 (36.4)	6.2 (19.4)	28.7 (36.5)	8.3 (21.5)	33.1 (37.8)	7.9 (19.9)	14.0 (27.9)	1.3 (8.8)
*PgR (categorical)*										
Negative (< 1%)	332 (37.2)	225 (82.4)	102 (41.3)	77 (84.6)	142 (43.7)	48 (71.6)	111 (39.6)	82 (75.2)	125 (69.1)	84 (95.5)
Positive (≥ 1%)	561 (62.8)	48 (17.6)	145 (58.7)	14 (15.4)	183 (56.3)	19 (28.4)	169 (60.4)	27 (24.8)	56 (30.9)	4 (4.5)
Ki-67 (continuous, 0%–100%)	37.9 (25.0)	62.9 (21.0)	38.4 (22.4)	58.5 (23.5)	38.6 (25.4)	62.0 (23.1)	37.5 (25.2)	58.1 (25.0)	52.0 (21.3)	66.4 (18.5)
*Ki-67 (categorical)*										
Negative (< 14%)	164 (18.4)	4 (1.5)	14 (5.7)	1 (1.1)	53 (16.3)	2 (3.0)	53 (18.9)	5 (4.6)	5 (2.8)	0 ﻿(0.0)
Positive (≥ 14%)	729 (81.6)	269 (98.5)	233 (94.3)	90 (98.9)	272 (83.7)	65 (97.0)	227 (81.1)	104 (95.4)	176 (97.2)	88 (100)
*Molecular subtype*^*c*^										
Luminal A–like	388 (43.4)	17 (6.2)	68 (27.5)	4 (4.4)	122 (37.5)	10 (14.9)	129 (46.1)	14 (12.8)	33 (18.2)	0﻿ (0.0)
Luminal B–like	279 (31.2)	64 (23.4)	109 (44.1)	21 (23.1)	104 (32.0)	21 (31.3)	82 (29.3)	28 (25.7)	35 (19.3)	7 (8.0)
Triple-negative	226 (25.3)	192 (70.3)	70 (28.3)	66 (72.5)	99 (30.5)	36 (53.7)	69 (24.6)	67 (61.5)	113 (62.4)	81 (92.0)
*Chemotherapy*^*d*^										
Anthracycline	673 (75.4)	131 (48.0)	208 (86.7)	71 (81.6)	325 (100.0)	67 (100.0)	280 (100.0)	109 (100.0)	181 (100.0)	88 (100.0)
Platinum	132 (14.8)	130 (47.6)	17 (7.1)	15 (17.2)	0 (0.0)	0 (0.0)	0 ﻿(0.0)	0 ﻿(0.0)	0 ﻿(0.0)	0 ﻿(0.0)
Taxane	721 (80.7)	248 (90.8)	228 (94.2)	86 (98.9)	325 (100.0)	67 (100.0)	280 (100.0)	109 (100.0)	181 (100.0)	88 (100.0)

Mean and SD are shown for continuous characteristics; frequency and percentage are shown for categorical characteristics

BMI, body mass index; ER, estrogen receptor (expression); ETC, high-dose epirubicin, taxane, and cyclophosphamide; HaBCS, Hannover breast cancer study; pCR, pathological complete response; PgR, progesterone receptor (expression); SD, standard deviation

^a^Complete information was available for 94.3% (n = 1100) of the patients. The percentage of missing values for each variable was below 0.5%, with the exception of BMI (2.8%) and grading (2.0%). Missing values were imputed as described above

^b^Missing values were substituted by best guesses (ie, median values, most common categories)

^c^Luminal A–like tumors are hormone receptor–positive (ER-positive or PgR-positive) with grade 1 or 2; luminal B–like tumors are hormone receptor–positive with grade 3; triple-negative tumors are ER-negative and PgR-negative

^d^The sum of the percentages is > 100 because some patients received combined chemotherapy

### Optimal cutoff points for biomarkers

The unadjusted relationship between ER, PgR, and Ki-67 and the pCR was best described as cubic spline functions with 1, 2, and 2 *df*, respectively (Supplementary Fig. S2a). The *df* obtained from cross-validation were confirmed using the AIC. The functions were monotonic and therefore enabled the determination of cut-off points. The optimal cut-off points for ER, PgR, and Ki-67 were 40%, 9%, and 30%, respectively (Supplementary Fig. S3).

### Comparison of pCR prediction models

The prediction model with linear usage of biomarkers (ER, PgR, Ki-67; 0% to 100% of positively stained cells) was most accurate (Table 2; cross-validated MSE, 0.1336). Additionally, all other models with continuous usage of the biomarkers were more accurate (0.1338–0.1347)—with decreasing accuracy correlating to increasing model complexity—than the model with established biomarker categories (0.1392) and the model with categories related to newly determined cutoff values (0.1356). These MSE results were confirmed by the AIC and AUC statistics (Table 2).

**Table 2:** Overall performance of the prediction models for pCR

Prediction model*	Apparent measure^a^			Cross-validated measure^b^	
	AIC	MSE	AUC	MSE	AUC
Null model	1271.1	0.1793	0.500	0.1797 (0.0094)	0.500 (0.000)
Basic model	1100.8	0.1525	0.759	0.1556 (0.0084)	0.748 (0.021)
Basic + ER, PgR, Ki-67 linear^c^	942.1	0.1292	0.845	0.1336 (0.0079)	0.836 (0.017)
Basic + ER, PgR, Ki-67 cubic spline, 2 *df*^c^	946.4	0.1291	0.846	0.1343 (0.0078)	0.833 (0.017)
Basic + ER, PgR, Ki-67 cubic spline, 3 *df*^c^	952.2	0.1290	0.846	0.1346 (0.0078)	0.832 (0.017)
Basic + ER, PgR, Ki-67 established categories	991.5	0.1350	0.825	0.1392 (0.0084)	0.814 (0.019)
Basic + ER, PgR, Ki-67 new categories^d^	948.1	0.1294	0.844	0.1356 (0.0080)	0.828 (0.018)

AIC, Akaike information criterion; AUC, area under the curve; *df,* degrees of freedom; ER, estrogen receptor (expression); MSE, mean squared error; pCR, pathological complete response; PgR, progesterone receptor (expression)

^*^The null model did not contain any predictors. The basic model included age at diagnosis, body mass index, tumor stage, grade, lymph node status, and tumor type. All other models were extensions of the basic model

^a^The models were fitted on the complete dataset. Confidence intervals were not calculated because model building and application to the same dataset might result in overoptimistic measures. A 95% confidence interval for the AUC would not then cover the true AUC with a 95% likelihood

^b^Summary statistics (mean and standard deviation in brackets) of MSE and AUC were obtained by threefold cross-validation with 100 repetitions

^c^Only three out of 27 continuous biomarker models are shown. These are the models with 1 (i.e. linear), 2, or 3 degrees of freedom for all three biomarkers, representing different levels of complexity

^d^The AIC is not a reliable measure for this model because it was applied to data that had already been used to identify the cutoff points

### The final prediction model

The regression coefficients of the final model and a formula for calculating a patient’s pCR-prob are presented in Table 3. Using an alternative definition of pCR gave similar results (Supplementary Table S2). The online calculator and the nomogram generate pCR-prob in a user-friendly format (see Supplement). Examples of ways in which biomarkers influence pCR-prob are shown in Supplementary Fig. S2b. Most patients in the primary study population had a low pCR-prob, particularly hormone receptor–positive patients (Supplementary Fig. S4). The final model was well calibrated (*p* = 0.90; Hosmer–Lemeshow χ^2^ test; Supplementary Fig. S5a). The apparent AUC was 0.009 units larger than the cross-validated AUC (0.845 versus 0.836, Table 2), indicating a small amount of overfitting. Cross-validated subgroup-specific AUC values were similar to the overall AUC for hormone-receptor positive (0.836) and ER-positive (0.834), but not for triple-negative breast cancer (TNBC) patients (0.662, Supplementary Table S4).

**Table 3:** The final logistic regression model for predicting pCR

Predictor		Coefficient (SE)	Odds ratio (95% CI)	*p* value
Intercept		− 1.9686 (0.9268)	–	–
Age (years)	Per year	− 0.0108 (0.0069)	0.99 (0.98–1.00)	0.12
100/BMI	Per 100 m^2^/kg	0.2951 (0.1141)	1.34 (1.07–1.68)	< 0.01
Tumor stage	Per stage	− 0.4824 (0.1212)	0.62 (0.49–0.78)	< 0.0001
Grade	Per grade	0.4206 (0.2274)	1.52 (0.98–2.38)	0.06
Lymph node status	cN0	0	1	–
	cN +	− 0.2430 (0.1701)	0.78 (0.56–1.09)	0.15
Tumor type	Other	0	1	–
	Ductal	− 0.1559 (0.1924)	0.86 (0.59–1.25)	0.42
	Lobular	− 0.4229 (0.5363)	0.66 (0.23–1.87)	0.43
ER	Per percent	− 0.0137 (0.0031)	0.99 (0.98–0.99)	< 0.0001
PgR	Per percent	− 0.0190 (0.0053)	0.98 (0.97–0.99)	< 0.01
Ki-67	Per percent	0.0170 (0.0044)	1.02 (1.01–1.03)	< 0.01

Regression coefficients with standard errors from the final regression model, associated odds ratios with 95% confidence intervals, and *p* values for Wald tests are shown. The *p* values and confidence intervals should be regarded as measures of importance within the regression model rather than measures of significance, especially since the conditions for statistical testing may not be fulfilled after a model selection process. The predicted probability *Prob* for pCR can be calculated using the following formula: Prob = exp(Z)/(1 + exp(Z)) with Z =  − 0.0190 + 0.9714 X and X =  − 1.9686 − 0.0108 age − 29.51/BMI − 0.4824 tumor stage + 0.4206 grade − 0.2430 cN − 0.1559 ductal − 0.4229 lobular − 0.0137 ER − 0.0190 PgR + 0.0170 Ki-67. Note that cN, ductal, and lobular values are one when present and zero when absent. 0.9714 is the shrinkage factor, and − 0.0190 is a correction term. Multiplying the result by 100 provides percentage values

BMI, body mass index; ER, estrogen receptor (expression); pCR, pathological complete response; PgR, progesterone receptor (expression); SE, standard error;  CI, confidence interval

The cross-validated ROC curve is shown in Supplementary Fig. S6. For cutoff points between 17 and 27%, sensitivities were between 0.80 and 0.89 at specificities of ≥ 0.65 (Supplementary Table S3). pCR-prob correlated moderately with IHC3 + C (ρ = 0.61, Supplementary Fig. S7).

**Fig. 2:**
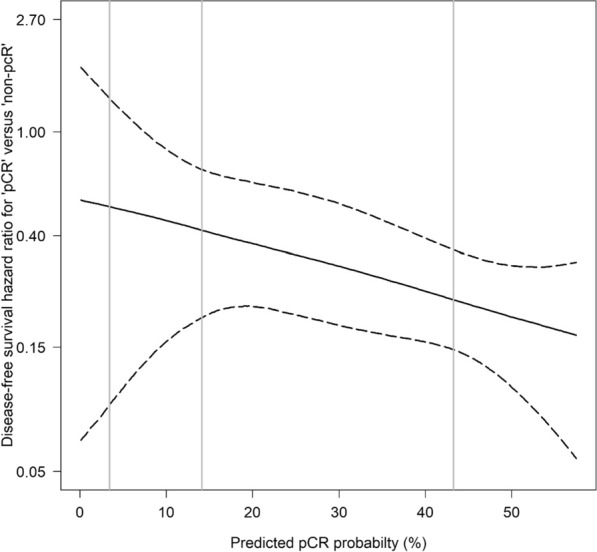
Disease-free survival HR for patients with pCR and patients without pCR (reference) as a continuous function of predicted pCR probabilities using data from the primary study population. The lower the value on the y axis, the higher the impact of pCR status on the prognosis. The solid curve shows mean HR values obtained from 20,000 random sample splittings. Dashed curves show the corresponding pointwise 95% central range (2.5th and 97.5th percentiles) of the HR distribution. The gray vertical lines indicate the first, second, and third quartiles of the predicted pCR probability in the primary study population. HR, hazard ratio; pCR, pathological complete response

A high pCR-prob not only indicated the efficacy of chemotherapy but also corresponded with the impact of pCR status on the prognosis. The greater the pCR-prob, the larger the benefit of a pCR in relation to DFS (Fig. 2). The hazard ratios for pCR presence vs absence improved from 0.55 (95% central range, 0.07–1.77) at a pCR-prob of 0% to 0.20 (0.11–0.31) at 50%.

**Fig. 3:**
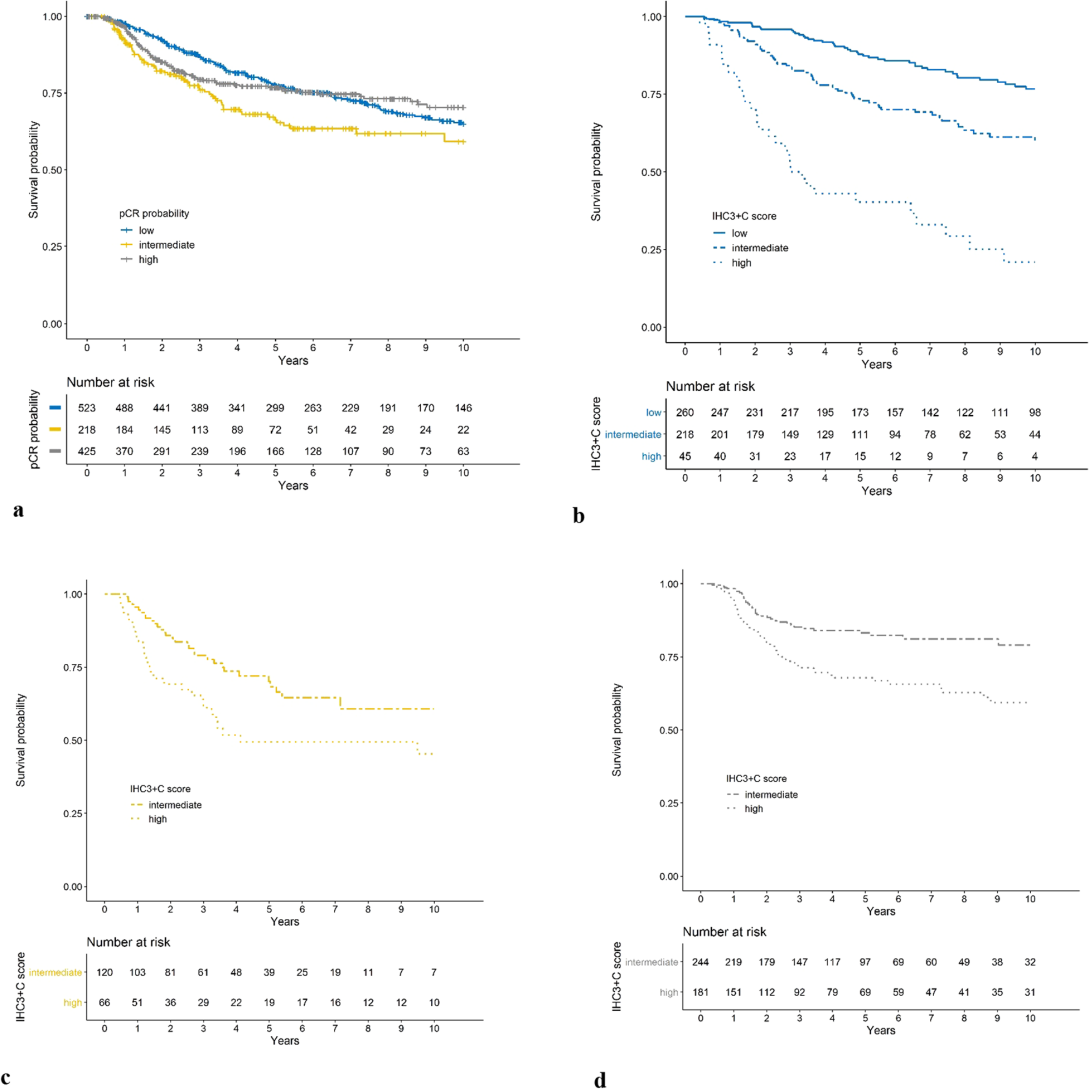
Kaplan–Meier estimates for disease-free survival relative to pCR probability classes (low, 0%–10%; intermediate, 10%–30%; high, 30%–100%) and IHC3 + C classes (low, < 210, first quartile; intermediate, 210–350, interquartile range; high, ≥ 350, third quartile): **a** for the complete primary study cohort, **b** for patients with a low pCR probability, **c** for patients with an intermediate pCR probability, and **d** for patients with a high pCR probability. The curves for low IHC3 + C and intermediate pCR are not shown because the sample size was small (n = 18). No patients had a low IHC3 + C with a high pCR. IHC3 + C = estrogen receptor, progesterone receptor, Ki-67, and clinical predictors; pCR, pathological complete response

Patients with a low pCR-prob (5-year DFS rate, 0.77; 95% CI, 0.74–0.81) and patients with a high pCR-prob (0.77; 0.72–0.81) had a better prognosis than patients with an intermediate pCR-prob (0.66; 0.59–0.74, Fig. 3a). This result was examined in more detail using the IHC3 + C score (Supplementary Fig. S8): The IHC3 + C score separated patients within patient groups defined by pCR-prob (Fig. 3b–d). Patients with a low pCR-prob and a high IHC3 + C value had an extraordinarily unfavorable DFS prognosis (10-year DFS rate, 0.21; 95% CI, 0.10–0.42) in comparison with patients with a high IHC3 + C but intermediate (0.45; 0.33–0.63) or a high pCR-prob (0.59; 0.51–0.70, Fig. 3b).

### External validation of the final prediction model

The AUC of the final prediction model was 0.827 (95% CI, 0.779–0.871) in the HaBCS. Sensitivities and specificities were similar to those in the primary cohort (Supplementary Table S3). The model was well calibrated (Supplementary Fig. S5b). The intercept and the slope of the calibration model were 0.26 (95% CI, –0.14–0.67) and 1.09 (95% CI, 0.77–1.42) and did not significantly differ from 0 and 1, respectively. Hence, updating of the model was not necessary.

The AUC was between 0.754 and 0.795 in the three analyzed treatment arms of GeparSepto and GeparOcto (Table 4). pCR-prob was satisfactorily precise in the GeparOcto-ETC arm, in patients with low pCR-prob in the GeparSepto-paclitaxel arm, and in those with high pCR-prob in the GeparSepto-NAB-paclitaxel arm. It was systematically too high and too low, respectively, for the other patients in the GeparSepto arms (Supplementary Fig. S5c–e), suggesting that the model should be recalibrated before application to data from GeparSepto-like treated patients (Table 4). The online calculator and the nomogram provide updated pCR-prob for such patients.

**Table 4:** Performance of the final prediction model for pCR in validation cohorts

	HaBCS	GeparSepto paclitaxel arm	GeparSepto nab-paclitaxel arm	GeparOcto ETC arm
AUC (95% CI)*	0.827 (0.779 to 0.871)	0.766 (0.704 to 0.822)	0.795 (0.746 to 0.840)	0.754 (0.695 to 0.811)
Calibration intercept (95% CI)^a^	0.30 (− 0.06 to 0.65)	− 0.78 (− 1.10 to − 0.47)	0.05 (− 0.25 to 0.35)	− 0.27 (− 0.56 to 0.02)
Calibration slope (95% CI)^a^	1.06 (0.79 to 1.33)	0.68 (0.46 to 0.90)	0.74 (0.55 to 0.92)	1.08 (0.70 to 1.46)
Model update necessary^b^	No	Yes	Yes	No

HaBCS, hannover breast cancer study; pCR, pathological complete response; AUC, area under the receiver operating characteristic curve

^*^ The 95% CI was estimated using 10,000 bootstrap samples

^a^95% CIs were calculated using regression coefficients and standard errors of the calibration model (logistic regression model)

^b^"Yes" means that, according to the specified criteria, the final prediction model needs to be recalibrated before it is applied to treatment arm–like patients. To recalibrate a model, replace the linear term X in the formula for the predicted pCR (see the footnote in Table 3) with the calibration intercept plus X multiplied by the calibration slope. For instance, replace X with − 0.78 + 0.68X for patients treated similarly to those in the GeparSepto paclitaxel arm. The AUC is not affected by recalibration

Subgroup-specific validations are shown in Supplementary Table S4. AUC values for hormone receptor-positive patients were between 0.789 and 0.810 in the validation cohorts, and the AUC values for TNBC patients (0.614 to 0.683) were similar to the value in the primary cohort.

## Discussion

This study developed and compared several prediction models for pCR after neoadjuvant chemotherapy in patients with HER2-negative BC, using clinical predictors and the molecular biomarkers ER, PgR, and Ki-67 assessed by IHC during routine clinical work. Integrating the molecular predictors as linear variables (0%–100%) yielded the best prediction of pCR. The validation of the prediction model in other populations yielded good results in patient groups with similar standard-of-care treatments. An online calculator and a nomogram are provided for user-friendly application of the prediction model.

To predict pCR, immunohistochemical markers were usually used categorically (positive vs negative), with predefined cutoff points that did not originate from pCR prediction [26, 27]. In the present study, the model with newly identified cutoff points performed better than the model with established cutoffs. However, the prediction model that did not use any cutoff points was favored overall.

With regard to prognosis, ER, PgR, and Ki-67 were also continuously incorporated into the IHC4 score in an adjuvant study on postmenopausal hormone receptor–positive patients [21]. The IHC4 score was also analyzed relative to predicting pCR after neoadjuvant chemotherapy [28, 29]. Good associations were found between IHC4 and pCR rates, but the AUC was 0.665 when IHC4 was combined with the Nottingham prognostic index [28]. Some multigene assays have also been analyzed in relation to predicting chemotherapy responsiveness, but none of these tests has been directly compared with classic IHC markers in a joint study, and the reported results do not indicate any superiority for the multigene tests [5].

pCR probability has not previously been considered in decision making for or against neoadjuvant chemotherapy. A survey among physicians with long-term experience showed that 84% would welcome the opportunity to use probability of pCR in decision making [3]. Using pCR-prob may be reasonable because the present study shows that the higher the pCR-prob, the greater the impact of pCR on the prognosis. This finding ensures not only consideration of the number of patients achieving a pCR but also the selection of a patient group with a greater prognostic benefit from achieving a pCR. We have shown that patients with high pCR-prob have a more favorable prognosis than patients with intermediate pCR-prob, which supports the view that patients with high pCR-prob can be treated with neoadjuvant chemotherapy.

A meta-analysis of randomized studies comparing neoadjuvant with adjuvant chemotherapy showed that the mortality rate in patients who do not respond to neoadjuvant chemotherapy was much higher (33.5% at 10 years) than in all patients who received adjuvant chemotherapy (22.7% at 10 years) [30]. This finding may largely reflect patient selection rather than a treatment effect [30]. Since the analyses were adjusted for clinical tumor size and nodal status, it might also indicate that some patients not only derive no benefit from neoadjuvant chemotherapy but also have prognoses that are poorer than expected. In the present study, this group of patients may correspond to patients with a low pCR-prob and a high risk of recurrence.

The most precise external prediction was obtained in a hospital-based cohort (HaBCS) that underwent preoperative chemotherapy very similar to that of the primary cohort (ie, an anthracycline-based agent followed by a taxane). The predictor showed good results in patients whose treatments were close to standard care (GeparSepto; NCT01583426; paclitaxel or nab-paclitaxel followed by anthracycline-based chemotherapy). The prediction model did not take treatments into account, and the significant treatment effect in GeparSepto might therefore have led to overestimated predictions in one arm and underestimated predictions in the other, necessitating correction by recalibration.

Differences in performance between the primary and validation cohorts are unavoidable and were expected [19]. More external validation studies that cover various geographic regions, time periods, and healthcare institutions may be able to further clarify such differences and possibly improve the prediction model. Adding other novel biomarkers, such as tumor-infiltrating lymphocytes, to the prediction model might also improve accuracy. The pCR prediction in patients with rare tumor types such as lobular breast cancer could be improved by using additional, sufficiently large specific cohorts.

The AUC values for hormone-receptor positive patients—i.e., patients for whom the use of the prediction tool is primarily intended—were around 0.80 and only slightly smaller than in the primary study population. The AUC values in TNBC patients were similar across all cohorts, however poorer (around 0.65) than in other patient groups, but still within the range of published studies. This may not be of great importance clinically as therapy decisions for chemotherapy in TNBC patients (as in HER2-positve patients) are usually made without considering further tumor-related characteristics because therapy efficacy is high and there is a lack of alternative treatment options.

The final prediction model was assessed both internally by cross-validation and externally in independent validation cohorts in accordance with the TRIPOD statement [19]. In both cases, prediction performance was measured with data not used for model building in order to obtain realistic results. Data splitting was also applied in survival analyses with actual and predicted pCR values in order to avoid biased predictions in which the upcoming event was already known. Both the AIC and cross-validation provided very similar model selection and model complexity results. It follows that using the AIC instead of cross-validation may be appropriate in complex settings. In the present study, the AIC rather than cross-validation was applied in the model development process to some extent in order to keep the number of potential models manageable.

## Conclusions

This study provides a prediction model for pCR after neoadjuvant chemotherapy using clinical parameters and continuous IHC markers. The good performance of the model suggests that therapy decisions could be based on predicted pCR probabilities. Considering both the likelihood of pCR and the prognosis not only identifies patients who may benefit from neoadjuvant chemotherapy but also patients in whom the prognosis was unexpectedly unfavorable. It may be necessary to develop novel treatment strategies for these patients.

## Supplementary Information

Additional file 1.

## Data Availability

For eligible studies, qualified researchers who provide a methodologically sound proposal and whose proposed use of data has been approved by a review committee may request access to individual patient data that underlie the results reported in this article, after de-identification (text, tables, figures, and appendices) and study protocols, beginning 9 months before and ending 36 months after article publication. Analyses to achieve the aims in the proposed proposal are eligible. Proposals may be submitted to the corresponding author up to 36 months following article publication. After 36 months, the data will be available in our university’s data warehouse but without investigator support. The online calculator developed from the final prediction model for pCR is available at https://www.pcrpredictor.org. The web page also provides an open-source application of the calculator as a downloadable file. The R code to determine the pCR probabilities is available on request from the corresponding author. No datasets were generated or analysed during the current study.

## Abbreviations

AIC: Akaike information criterion
AUC: Area under the receiver operating characteristic curve
BC: Breast cancer
BMI: Body mass index
BSA: Body surface area
CI: Confidence interval
DFS: Disease-free survival
ER: Estrogen receptor
ERNEST-B: Erlangen neoadjuvant study breast
ETC: High-dose epirubicin, taxane, and cyclophosphamide
HaBCS: Hannover breast cancer study
HER2: Human epidermal growth factor receptor 2
iddEPC: Intense dose-dense epirubicin, paclitaxel, and cyclophosphamide
IHC: Immunohistochemical
IHC3 + C: IHC 3 score plus clinical predictors
IQR: Interquartile range
MSE: Mean squared error
pCR: Pathological complete response
pCR-prob: Probability of pathological complete response
PgR: Progesterone receptor
PM[Cb]: Paclitaxel plus nonpegylated liposomal doxorubicin with additional carboplatin
ROC: Receiver operating characteristic
SD: Standard deviation
SE: Standard error
