# Supplementary material for: Prediction of pathological complete response after neoadjuvant chemotherapy for HER2-negative breast cancer patients with routine immunohistochemical markers
Source: Breast Cancer Res. 2025 Jan 24;27:13. doi: 10.1186/s13058-025-01960-8 (PMC11759445; doi:10.1186/s13058-025-01960-8)

Supplementary material for:

Prediction of pathological complete response after neoadjuvant chemotherapy for HER2-negative breast cancer patients with routine immunohistochemical markers

Lothar Häberle^1,2^, Ramona Erber^3^, Paul Gass^1^, Alexander Hein^1^, Melitta Niklos^1^, Bernhard Volz^1,4^, Carolin C. Hack^1^, Rüdiger Schulz-Wendtland^5^, Hanna Huebner^1^, Chloë Goossens^1^, Matthias Christgen^6^, Thilo Dörk^7^, Tjoung-Won Park-Simon^8^, Andreas Schneeweiss^9^, Michael Untch^10^, Valentina Nekljudova^11^, Sibylle Loibl^11^, Arndt Hartmann^3^, Matthias W. Beckmann^1^, Peter A. Fasching^1^

^1^ Department of Gynecology and Obstetrics, Erlangen University Hospital, Comprehensive Cancer Center Erlangen-EMN, Friedrich Alexander University of Erlangen–Nuremberg, Erlangen, Germany

^2^ Biostatistics Unit, Department of Gynecology and Obstetrics, Erlangen University Hospital, Erlangen, Germany

^3^ Institute of Pathology, Erlangen University Hospital, Comprehensive Cancer Center Erlangen-EMN, Friedrich Alexander University of Erlangen–Nuremberg, Erlangen, GermKany

^4^ Ansbach University of Applied Sciences, Ansbach, Germany

^5^ Institute of Diagnostic Radiology, Erlangen University Hospital, Comprehensive Cancer Center Erlangen-EMN, Friedrich Alexander University of Erlangen–Nuremberg, Erlangen, Germany

^6^ Institute of Pathology, Hannover Medical School, Hannover, Germany

^7^ Gynecology Research Unit, Hannover Medical School, Hannover, Germany

^8^ Department of Gynecology and Obstetrics, Hannover Medical School, Hannover, Germany

^9^ National Center for Tumor Diseases, University Hospital and German Cancer Research Center, Heidelberg, Germany

^10^ Department of Gynecology and Obstetrics, Helios Clinic Berlin-Buch, Berlin, Germany

^11^ German Breast Group, Neu-Isenburg, Germany

**SUPPLEMENTARY METHODS**

## **Primary Study Population**

The patients in the single-center Erlangen Neoadjuvant Study Breast (ERNEST-B) were selected from a consecutive cohort of patients who were diagnosed with breast cancer and treated with neoadjuvant chemotherapy at the University Breast Center for Franconia (Erlangen, Germany) between 2002 and 2020. During that period, 9052 patients with primary invasive breast cancer were treated, 2034 (22.5%) of whom received neoadjuvant chemotherapy. Neoadjuvant chemotherapy was indicated during clinical routine work in accordance with national and international guidelines. For inclusion in the present study, the patients had to be ≥18 years old and have undergone surgery following neoadjuvant chemotherapy (*n* = 2005). Male patients (*n* = 1), patients with metastases (*n* = 141) or contralateral breast cancer at primary diagnosis (*n* = 105), patients with an HER2-positive tumor (*n* = 493), and patients with incomplete information for estrogen receptor (ER), progesterone receptor (PgR), or Ki-67 (*n* = 89) were excluded, resulting in a final sample size of 1166 patients. Approval for the analyses was obtained from the ethics committee of the University of Erlangen–Nuremberg (No. 297 17 BC).

Patients with triple-negative disease were not excluded, although they are known to have high pCR rates and a good disease-free survival prognosis when a pCR is achieved.^1^ These patients were included because the present study was aimed at developing prediction models that use ER and PgR continuously, each with values from 0% to 100%. Valid pCR predictions for patients with ER=0% and PgR=0% or values that are close to zero — i.e., patients with “almost triple-negative” disease — are thus made possible.

Adjuvant radiotherapy and systemic therapy were administered in accordance with national guidelines.^2^ Adjuvant chemotherapy was administered in 178 (15.3%) of the 1166 patients. They comprised 16 (5.9%) of 273 patients who achieved a pCR and 162 (18.1%) of 893 patients who did not achieve a pCR. A total of 875 (75.0%) patients were treated with adjuvant radiotherapy: 189 (69.3%) of those who achieved a pCR and 686 (76.8%) of those who did not. The corresponding figures for adjuvant endocrine therapy are 642 (55.1%), 54 (19.8%), and 588 (65.9%), respectively.

The University Breast Center for Franconia has been certified by the German Cancer Society (Deutsche Krebsgesellschaft) and the German Society for the Study of Breast Diseases (Deutsche Gesellschaft für Senologie). To obtain certification, the Center must document each breast cancer case in a prospective database, including patient and tumor characteristics, detailed treatment, and epidemiological data. As part of the certification process, compliance of treatment decisions with German guidelines for the treatment of breast cancer were checked. Follow-up data were collected for ≤10 years after the primary diagnosis. The documentation quality was audited annually as part of the continuous certification process.

## **Immunohistochemical Staining**

Monoclonal mouse/rat antibodies against ER-α (before 2014: clone 1D5 1:200 dilution; since 2014: clone EP1, 1:40 dilution [Dako Denmark, Glostrup, Denmark]), monoclonal mouse antibody against the PgR (before November 2018: clone pgR636, 1:200 dilution [Dako Denmark]; since November 2018: PR, clone 1E2 [Ventana ready-to-use assay; Roche Diagnostics, Basel, Switzerland]), and monoclonal antibody against Ki-67 (clone MIB-1, 1:200 dilution before 2015 and 1:100 dilution since 2015 [Dako Denmark]) were used to stain the preoperative core biopsies. The percentage of positively stained tumor cells was mentioned in the pathology reports. A polyclonal antibody against HER2 (1:200 dilution before 2012 and 1:1000 dilution since 2012 [Dako Denmark]) was used, and HER2 status was documented in the pathology reports as negative, 0, 1+, 2+, or 3+ in accordance with published guidelines.^3^ Tumors with a score of 0 or 1+ were defined as HER2-negative, and those with a score of 3+ were regarded as HER2-positive. Tumors with a 2+ staining were tested for gene copy numbers of HER2 using chromogene in situ hybridization. The gene copy numbers of HER2 and the centromeres of the corresponding chromosome 17 were stained using a kit with two probes of different colors (ZytoDot, 2C SPEC HER2/CEN17 [ZytoVision GmbH, Bremerhaven, Germany]). A tumor was regarded as HER2-positive if the HER2/CEN17 ratio was ≥2.2 before 2013 and ≥2 thereafter.^4^

## **Univariable Statistical Analyses**

The biomarkers ER, PgR, and Ki-67 were regarded as continuous variables (0%–100% of positively stained cells). The possibly nonlinear relationship between a biomarker and pCR (yes vs no) without consideration of any covariates was described using logistic regression models with natural cubic spline functions.^5^ The degrees of freedom (*df*; 1–3) were each determined by cross-validation using the mean squared error (MSE) as the evaluation criterion. For sensitivity analysis, *df* were determined using the Akaike information criterion (AIC), as was done in Salmen et al.^6^ Optimal cutoff values for ER, PgR, and Ki-67 were calculated in relation to pCR status using the minimum *P* value approach in logistic regression analyses.^7,8^

## **Validation Cohorts**

The Hannover Breast Cancer Study (HaBCS) was originally started as a hospital-based case-control study, with recruitment from 1996 to 2001, and was continued in a second stage as an observational hospital-based cohort study with recruitment from 2011 to 2021. The present study included female patients with breast cancer who were treated for a HER2-negative tumor with neoadjuvant chemotherapy between 2011 and 2020 at four certified breast cancer centers in the Hannover region, were ≥18 years old, and had information available about pCR status from the final surgery (*n* = 413). Patients with metastases (*n* = 29) or contralateral breast cancer at diagnosis (*n* = 8), and patients with incomplete ER, PgR, Ki-67, age, or tumor stage information (*n* = 46; see Table 1, footnote 3) were excluded, resulting in a final sample size of 338 patients. Approval for the analyses was obtained from the ethics committee at Hannover Medical School (No. 6079). Adjuvant radiotherapy and systemic therapy were administered in accordance with national guidelines. Adjuvant chemotherapy was administered in 46 (13.6%) of the 338 patients. They comprised two (2.2%) of 91 patients who achieved a pCR and 44 (17.9%) of 247 patients who did not achieve a pCR. A total of 315 (93.2%) patients were treated with adjuvant radiotherapy: 81 (88.6%) of those who achieved a pCR and 234 (94.7%) of those who did not. The corresponding figures for adjuvant endocrine therapy are 199 (58.6%), 19 (21.1%), and 180 (72.7%), respectively.

GeparSepto and GeparOcto were each multicenter, prospective, randomized, phase 3 trials for neoadjuvant treatment of high-risk early breast cancer. Women with newly diagnosed primary invasive breast cancer were enrolled after providing written informed consent for study participation and central histology evaluation of core biopsies. Both studies were approved by the appropriate ethics committees and institutional review boards and the competent authority.

GeparSepto was a trial to compare nanoparticle albumin-bound (nab-)paclitaxel vs solvent-based (sb) paclitaxel. The tumor had to be cT2 to cT4 or cT1 with an additional high-risk factor: either clinically or pathologically node-positive, hormone receptor–negative, HER2-positive, or with Ki-67 >20%. A total of 1206 patients started neoadjuvant treatment, 606 with nab-paclitaxel and 600 with sb-paclitaxel. Postoperative radiotherapy and systemic treatments were given in accordance with standard German guidelines.^9,10^

For the present study, patients with a HER-negative tumor were included (*n =* 810). Patients with contralateral breast cancer at diagnosis (*n* = 17), or missing surgery data (*n* = 12) were excluded, resulting in a final sample size of 781 patients.

GeparOcto was a trial to compare two dose-dense regimens: intense dose-dense epirubicin, paclitaxel, and cyclophosphamide (iddEPC; the ETC arm) vs paclitaxel plus nonpegylated liposomal doxorubicin with additional carboplatin in triple-negative breast cancer (the PM[Cb] arm). The tumor had to be cT1c to cT4 and HER2-positive or triple-negative or luminal B-like (defined as hormone receptor–positive and Ki-67 >20%) and node-positive. A total of 945 patients started neoadjuvant treatment, 479 with iddEPC and 706 with PM(Cb). Adjuvant radiotherapy and systemic treatments were administered in accordance with national guidelines.^11,12^

For the present study, only the ETC arm was analyzed. The PM(Cb) arm was not analyzed because treatment differed between patient groups. Patients with triple-negative disease were treated differently from hormone receptor–positive patients.

Patients with a HER2-negative tumor were included (*n* = 281). Patients with contralateral breast cancer at diagnosis (*n* = 10), incomplete IHC biomarker information (*n* = 1), or missing surgery data (*n* = 1) were excluded, resulting in a final sample size of 269 patients.

Recruitment periods, detailed neoadjuvant treatment descriptions, and for purposes of comparison, the pCR rates in the primary and validation cohorts are presented in **Supplementary Table S1**.

## **Online Calculator**

The online calculator pCR Predictor is available at <https://www.pcrpredictor.org>. The web page also provides an open-source application of the calculator as a downloadable file. The formula for predicting pCR presented in the footnote of **Table 3** in this paper has been implemented in the online calculator.


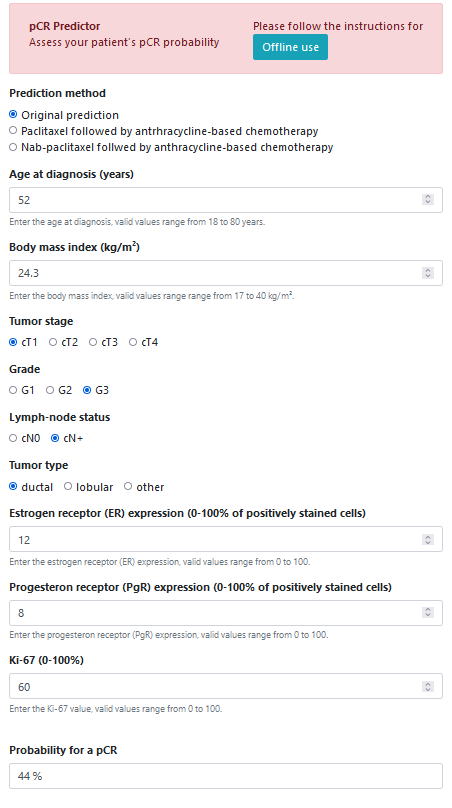


## **Nomogram**

The nomogram for predicting pCR is used in the following way: Locate the patient’s age at diagnosis on the Age axis. Draw a line straight upwards to the Points axis to determine how many points the patient receives. Repeat this process for each predictor, then add the points received for each predictor. Locate this total on the Total Points axis, and draw a line straight down to find the patient’s probability for achieving a pCR. If the prediction should be based on the original prediction model, find the value for pCR prob. (original). If preoperative therapy similar to that used in the GeparSepto study (paclitaxel or nab-paclitaxel followed by anthracycline-based chemotherapy) is available for the patient, find the value for pCR prob. (paclitaxel) or pCR prob. (nab-paclitaxel), as appropriate.


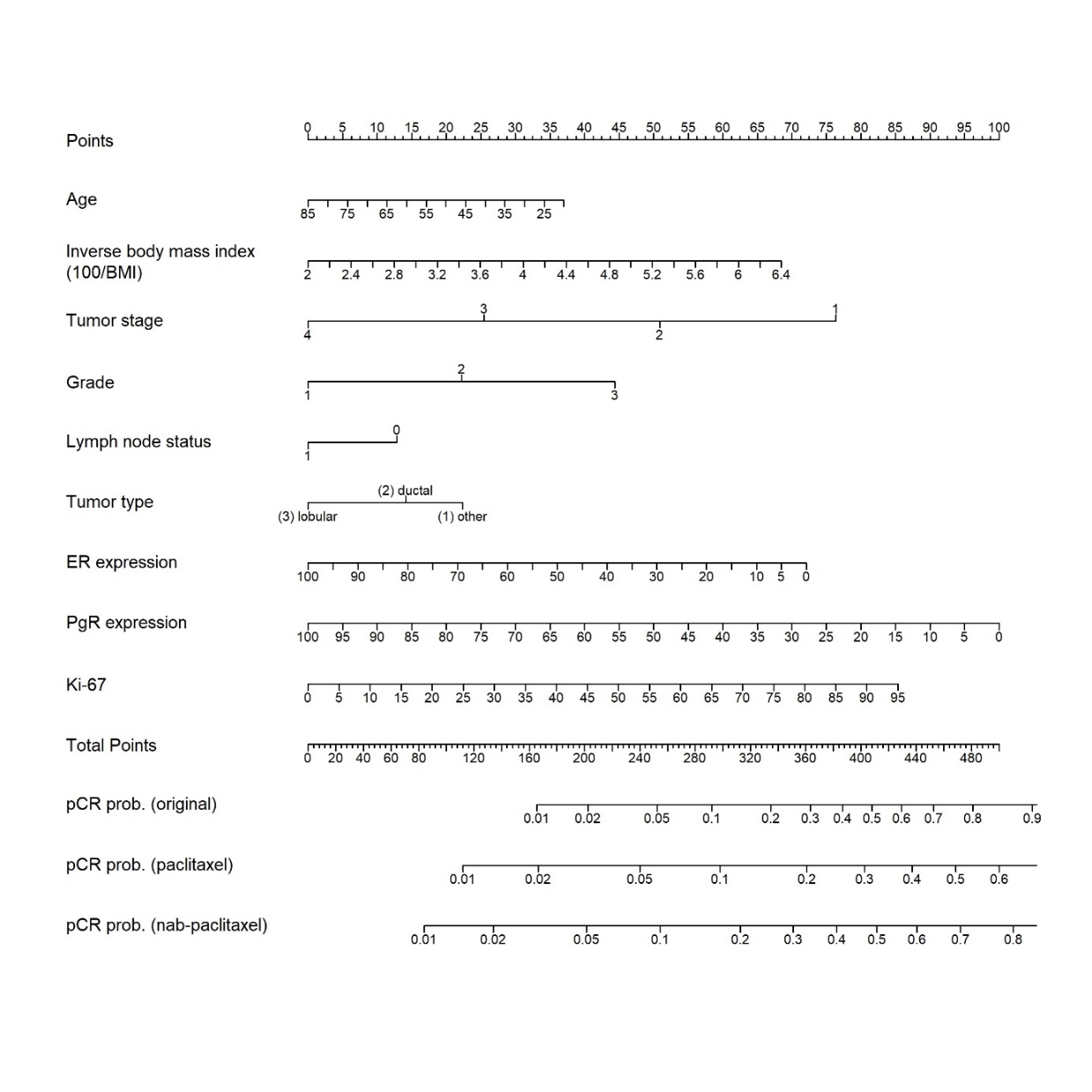


## **Case-Based Examples**

Two examples of the way in which the pCR probability calculator or the nomogram may be helpful for therapy decisions are presented below.

*Example 1*. A 52-year-old woman with a BMI of 24.3 kg/m^2^ is diagnosed with breast cancer. Her tumor is clinically classified as cT1, G3, cN+, and ductal. Immunohistochemical assessment gives 12%, 8%, and 60% positively stained cells for ER, PgR, and Ki-67, respectively. Inserting these values into the calculator online or the formula in **Table 3**, or using the nomogram, yields an estimated pCR probability of 44%. The clinician who treats this patient decides in favor of neoadjuvant chemotherapy. This clinician usually uses a pCR probability of 25% as the threshold for therapy decisions and treats patients with an estimated pCR probability above 25% with neoadjuvant chemotherapy, provided there is no reason not to use it. The threshold of 25% was chosen because the chemotherapy regimen is similar to that of the primary cohort and HaBCS; therefore, a sensitivity of approximately 80% and a specificity of about 70% (**Supplementary** **Table S3**) appear reasonable to the clinician.

*Example 2*. A 68-year-old patient with a BMI of 27.2 kg/m^2^, whose breast cancer is classified as cT2, G3, cN0, and lobular and at immunohistochemical assessment yields 15% ER, 10% PgR, and 45% Ki-67 positively stained cells, is assessed by the same clinician. In this case, the clinician decides against neoadjuvant chemotherapy because the predicted pCR probability is 21%.

**SUPPLEMENTARY REFERENCES**

1. von Minckwitz G, Untch M, Blohmer JU, et al. Definition and impact of pathologic complete response on prognosis after neoadjuvant chemotherapy in various intrinsic breast cancer subtypes. *J Clin Oncol* 2012; **30:** 1796–804.

2. Arbeitsgemeinschaft für Gynäkologische Onkologie. Kommission Mamma: diagnosis and treatment of patients with primary and metastatic breast cancer. Article in German. Accessed May 17, 2022. https://www.ago-online.de/leitlinien-empfehlungen/leitlinien-empfehlungen/kommission-mamma

3. Sauter G, Lee J, Bartlett JMS, et al. Guidelines for human epidermal growth factor receptor 2 testing: biologic and methodologic considerations. *J Clin Oncol* 2009; **27;** 1323–33.

4. Wolff AC, Hammond ME, Hicks DG, et al. Recommendations for human epidermal growth factor receptor 2 testing in breast cancer: American Society of Clinical Oncology/College of American Pathologists clinical practice guideline update. *J Clin Oncol* 2013; **31:** 3997–4013.

5. Harrell FE Jr, Lee KL, Pollock BG. Regression models in clinical studies: determining relationships between predictors and response. J Natl Cancer Inst 1988; **80:** 1198–202.

6. Salmen J, Neugebauer J, Fasching, PA, et al. Pooled analysis of the prognostic relevance of progesterone receptor status in five German cohort studies. *Breast Cancer Res Treat* 2014; **148:** 143–51.

7. Fasching PA, Heusinger K, Haeberle L, et al. Ki67, chemotherapy response, and prognosis in breast cancer patients receiving neoadjuvant treatment. *BMC Cancer* 2011; **11:** 486.

8. Mazumdar M, Glassman JR. Categorizing a prognostic variable: review of methods, code for easy implementation and applications to decision-making about cancer treatments. *Stat Med* 2000; **19:** 113–32.

9. Untch M, Jackisch C, Schneeweiss A, et al. Nab-paclitaxel versus solvent-based paclitaxel in neoadjuvant chemotherapy for early breast cancer (GeparSepto-GBG 69): a randomised, phase 3 trial. *Lancet Oncol* 2016; **17:** 345–56.

10. Untch M, Jackisch C, Schneeweiss A, et al. NAB-Paclitaxel improves disease-free survival in early breast cancer: GBG 69-GeparSepto. *J Clin Oncol* 2019; **37:** 2226–34.

11. Schneeweiss A, Mobus V, Tesch H, et al. Intense dose-dense epirubicin, paclitaxel, cyclophosphamide versus weekly paclitaxel, liposomal doxorubicin (plus carboplatin in triple-negative breast cancer) for neoadjuvant treatment of high-risk early breast cancer (GeparOcto-GBG 84): a randomised phase III trial. *Eur J Cancer* 2019; **106:** 181–92.

12. Schneeweiss A, Michel LL, Mobus V, et al. Survival analysis of the randomised phase III GeparOcto trial comparing neoadjuvant chemotherapy of intense dose-dense epirubicin, paclitaxel, cyclophosphamide versus weekly paclitaxel, liposomal doxorubicin (plus carboplatin in triple-negative breast cancer) for patients with high-risk early breast cancer. *Eur J Cancer* 2022; **160:** 100–11.

**SUPPLEMENTARY TABLES**

Supplementary Table S1: **Description of the primary study cohort and validation cohorts**

| **Cohort** | **Recruitment** | **Neoadjuvant treatment** | **pCR rate (%)*** |
| --- | --- | --- | --- |
| ERNEST (primary cohort) | 2002–2020 | Anthracycline, platinum, taxane (see Table 1) | 23.4 |
| HaBCS | 2011–2021 | Mostly anthracycline and taxane (see Table 1) | 26.9 |
| GeparSepto: paclitaxel arm | 2012–2013 | 12 × T(80) weekly → 4 × E(90) C(600) q3w^†^ | 17.1 |
| GeparSepto: nab-paclitaxel arm | 2012–2013 | 12 × nP (125) weekly → 4 × E(90)/C(600) q3w^†^ | 28.0 |
| GeparOcto: ETC arm | 2014–2016 | 3 × E(150) → 3 × T(225) → 3 × C(2000)^†^ | 32.7 |

BSA=body surface area; HaBCS=Hannover Breast Cancer Study; T=paclitaxel; E=epirubicin; nP=nab-paclitaxel; C=cyclophosphamide.

* Observed pCR rate in each cohort.

^†^ Doses are per m^2^ BSA.

Supplementary Table S2: **Final logistic regression model for predicting pCR using an alternative definition of pCR: no invasive tumor cells in the breast and no tumor cells in the lymph nodes; noninvasive tumor cells in the breast allowed (ypT0/is, ypN0)***

| **Predictor** |  | **Coefficient (SE)** | **Odds ratio (95% CI)** | ***P* value** |
| --- | --- | --- | --- | --- |
| Intercept |  | −1.3127 (0.8759) | .. | .. |
| Age (years) | Per year | −0.0187 (0.0068) | 0.98 (0.97–0.99) | <0.01 |
| 100/BMI | Per 100 m^2^/kg | 0.3418 (0.1110) | 1.41 (1.13–1.75) | <0.01 |
| Tumor stage | Per stage | −0.4573 (0.1155) | 0.63 (0.50–0.79) | <0.0001 |
| Grade | Per grade | 0.3967 (0.2097) | 1.49 (0.99–2.24) | 0.06 |
| Lymph node status | cN0 | 0 | 1 | .. |
|  | cN+ | −0.3077 (0.1647) | 0.74 (0.53–1.02) | 0.06 |
| Tumor type | Other | 0 | 1 | .. |
|  | Ductal | −0.2856 (0.1873) | 0.75 (0.52–1.08) | 0.13 |
|  | Lobular | −0.6162 (0.4961) | 0.54 (0.20–1.43) | 0.21 |
| ER | Per unit | −0.0129 (0.0028) | 0.99 (0.98–0.99) | <0.00001 |
| PgR | Per unit | −0.0171 (0.0045) | 0.98 (0.97–0.99) | <0.001 |
| Ki-67 | Per unit | 0.0163 (0.0043) | 1.02 (1.01–1.02) | <0.001 |

AUC=area under the curve; BMI=body mass index; ER=estrogen receptor (expression); ETC=high-dose epirubicin, taxane, and cyclophosphamide; haBCS=Hannover Breast Cancer Study; pCR=pathological complete response; PgR=progesterone receptor (expression); SE=standard error; CI confidence interval.

* Regression coefficients with standard errors, odds ratios with 95% confidence intervals, and *P* values for Wald tests are shown. In all, 316 (27.1%) of 1166 patients achieved a pCR (ypT0/is, ypN0). The predicted probability for pCR (ypT0/is, ypN0) can be calculated with the formula for the final prediction model in the main manuscript after substituting the regression coefficients, the shrinkage factor (0.9738), and the correction term (−0.0144). This alternative prediction model performed similarly to the main model. The apparent AUC in the primary study population was 0.845, the cross-validated AUC was 0.837 (standard deviation, 0.016) and the AUCs in the external validation cohorts were as follows: HaBCS, 0.807 (95% CI, 0.756−0.856); GeparSepto paclitaxel arm, 0.752 (95% CI, 0.696−0.806); GeparSepto nab-paclitaxel arm, 0.755 (95% CI, 0.701−0.804); GeparOcto ETC arm, 0.770 (95% CI, 0.712−0.823).

Supplementary Table S3**: Sensitivity and specificity of the final prediction model for pCR* in the primary study cohort and in validation cohorts for decision making for or against neoadjuvant chemotherapy**

|  |  | **Primary study cohort^§^** | | **HaBCS** | | **GeparSepto paclitaxel arm** | | | **GeparSepto nab-paclitaxel arm** | | | **GeparOcto ETC arm** | |
| --- | --- | --- | --- | --- | --- | --- | --- | --- | --- | --- | --- | --- | --- |
| **Cutoff point^†^** | **Frequency above cutoff point, %^‡^** | **Sensitivity** | **Specificity** | **Sensitivity** | **Specificity** | **Updated cutoff^¶^** | **Sensitivity** | **Specificity** | **Updated cutoff^¶^** | **Sensitivity** | **Specificity** | **Sensitivity** | **Specificity** |
| 10 | 55.1 | 0.93 | 0.56 | 0.93 | 0.55 | 9.4 | 0.91 | 0.46 | 17.2 | 0.88 | 0.55 | 0.99 | 0.23 |
| 12 | 52.0 | 0.92 | 0.59 | 0.92 | 0.59 | 10.7 | 0.91 | 0.50 | 19.4 | 0.85 | 0.56 | 0.99 | 0.25 |
| 15 | 49.1 | 0.90 | 0.63 | 0.91 | 0.62 | 12.5 | 0.87 | 0.53 | 22.6 | 0.85 | 0.60 | 0.99 | 0.28 |
| 17 | 47.6 | 0.89 | 0.65 | 0.89 | 0.64 | 13.7 | 0.87 | 0.55 | 24.5 | 0.83 | 0.62 | 0.98 | 0.30 |
| 20 | 44.4 | 0.86 | 0.68 | 0.89 | 0.65 | 15.3 | 0.85 | 0.58 | 27.4 | 0.81 | 0.65 | 0.97 | 0.33 |
| 22 | 43.0 | 0.85 | 0.70 | 0.89 | 0.66 | 16.4 | 0.85 | 0.59 | 29.2 | 0.80 | 0.66 | 0.97 | 0.34 |
| 25 | 40.9 | 0.82 | 0.72 | 0.81 | 0.69 | 18.0 | 0.79 | 0.62 | 31.8 | 0.78 | 0.69 | 0.95 | 0.36 |
| 27 | 38.8 | 0.80 | 0.74 | 0.80 | 0.72 | 19.1 | 0.76 | 0.64 | 33.4 | 0.77 | 0.69 | 0.95 | 0.39 |
| 30 | 36.4 | 0.77 | 0.76 | 0.77 | 0.76 | 20.7 | 0.73 | 0.69 | 35.9 | 0.73 | 0.73 | 0.93 | 0.44 |
| 32 | 35.2 | 0.75 | 0.77 | 0.76 | 0.78 | 21.8 | 0.72 | 0.70 | 37.5 | 0.72 | 0.75 | 0.89 | 0.46 |
| 35 | 32.6 | 0.72 | 0.80 | 0.71 | 0.81 | 23.4 | 0.64 | 0.72 | 39.9 | 0.70 | 0.77 | 0.85 | 0.48 |
| 40 | 28.3 | 0.65 | 0.84 | 0.56 | 0.85 | 26.1 | 0.51 | 0.78 | 43.7 | 0.61 | 0.82 | 0.76 | 0.60 |

AUC=area under the curve; ER=estrogen receptor (expression); ETC=high-dose epirubicin, taxane, and cyclophosphamide; HaBCS=Hannover Breast Cancer Study; pCR=pathological complete response; pCR-prob=pCR probability; PgR=progesterone receptor (expression).

* Logistic regression model with the predictors age at diagnosis, body mass index, tumor stage, grade, lymph node status, and tumor type and the continuous biomarkers ER, PgR, and Ki-67.

^†^ Patients were classified into a high-pCR–probability group if the prediction model assigned a pCR-prob above the cutoff point; patients were classified into a low-pCR–probability group otherwise. Sensitivity (between 0 and 1) is defined as the proportion of high-pCR–probability patients among those with an observed pCR. Specificity (between 0 and 1) is defined as the proportion of low-pCR–probability patients among those without an observed pCR.

^‡^ The proportion of patients classified as high-probability in the primary study population.

^§^ Measurements were obtained by threefold cross-validation with 100 repetitions.

According to prespecified criteria, the final prediction model needs to be recalibrated before application to patients treated similarly to those in the GeparSepto study in order to obtain precise predictions (see **Table 4**). Recalibration does not affect sensitivity, specificity, or the AUC. The cutoff points, however, must be adjusted when an updated model is used. For instance, sensitivity and specificity are 0.91 and 0.46 for patients treated similarly to those in the GeparSepto paclitaxel arm when the original prediction model, together with a cutoff point of 10%, is used for decision making. Sensitivity and specificity remain the same (0.91 and 0.46) when the updated prediction model with cutoff point 9.4% is used instead.

Supplementary Table S4**: The area under the receiver operating characteristic curve (AUC) for different patient subgroups in the primary study population and validation cohorts, using the final prediction model**

|  | Primary Study Population | | HaBCS | | GeparSepto Paclitaxel | | GeparSepto Nab-paclitaxel | | GeparOcto ETC | |
| --- | --- | --- | --- | --- | --- | --- | --- | --- | --- | --- |
| Subgroup | N | AUC cross-v.^1^ | N | AUC (95% CI)^2^ | N | AUC (95% CI) ^2^ | N | AUC (95% CI) ^2^ | N | AUC (95% CI) ^2^ |
| all patients^3^ | 1166 | 0.836 | 338 | 0.827 (0.779, 0.871) | 392 | 0.766 (0.704, 0.822) | 389 | 0.795 (0.746, 0.840) | 269 | 0.754 (0.695, 0.811) |
| HR-positive | 748 | 0.836 | 202 | 0.810 (0.708, 0.896) | 257 | 0.804 (0.713, 0.882) | 253 | 0.789 (0.705, 0.865) | 75 | 0.804 (0.640, 0.928) |
| ER-positive | 721 | 0.834 | 191 | 0.769 (0.648, 0.877) | 235 | 0.786 (0.668, 0.886) | 235 | 0.755 (0.655, 0.845) | 69 | 0.829 (0.645, 0.956) |
| TNBC | 418 | 0.662 | 136 | 0.680 (0.590, 0.768) | 135 | 0.645 (0.529, 0.756) | 136 | 0.614 (0.519, 0.707) | 194 | 0.683 (0.605, 0.757) |

CI=confidence interval; HR=hormone receptor; ER=estrogen receptor; TNBC=triple-negative breast cancer.

^1^ AUC values for the primary study population were obtained by 3-fold cross-validation with 100 repetitions. In cross-validation, the complete dataset was split into training and validation datasets, and the final prediction model was fitted to the training set and then applied to the validation set restricted to a subgroup.

^2^ The final prediction model was applied to the validation cohort restricted to a patient subgroup, and the AUC was calculated. The confidence interval was estimated using 10,000 bootstrap samples.

^3^ Results for the complete cohort are shown for the purpose of comparison with subgroup-specific results.

**SUPPLEMENTARY FIGURES**

Supplementary Figure S1: **Distribution of the assessed biomarkers in the primary study population.** (**a**) ER expression, (**b**) PgR expression, and (**c**) Ki-67. [ER=estrogen receptor; PgR=progesterone receptor]

| **a**  **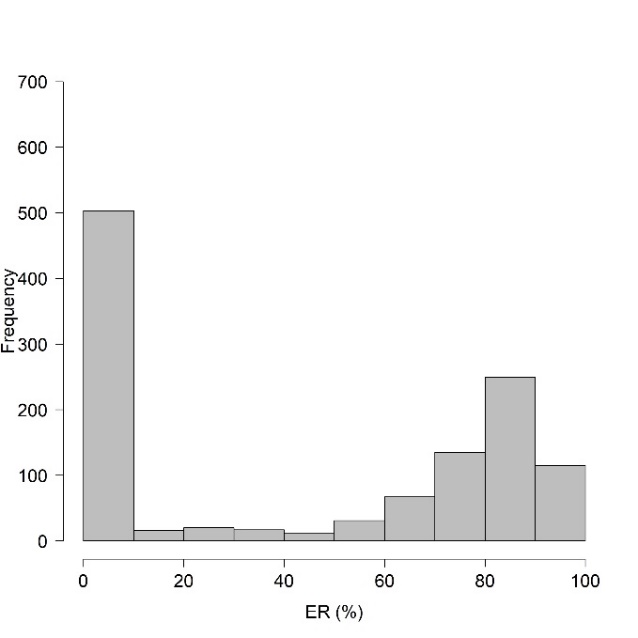** | **b**  **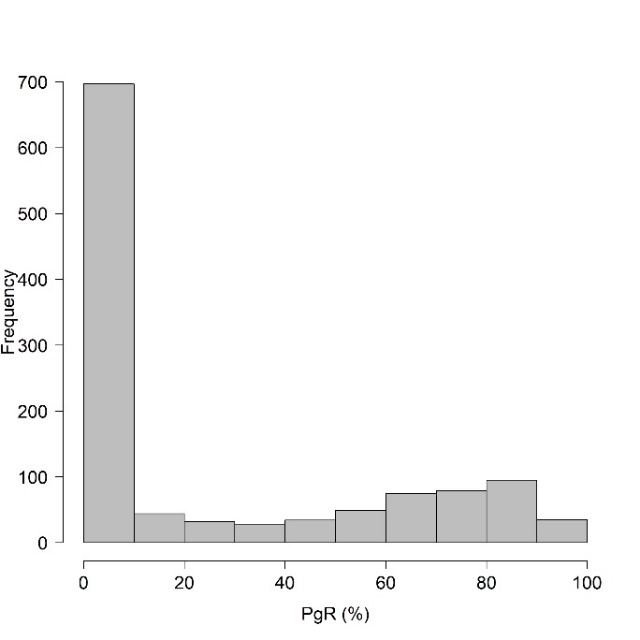** |
| --- | --- |
| **c**  **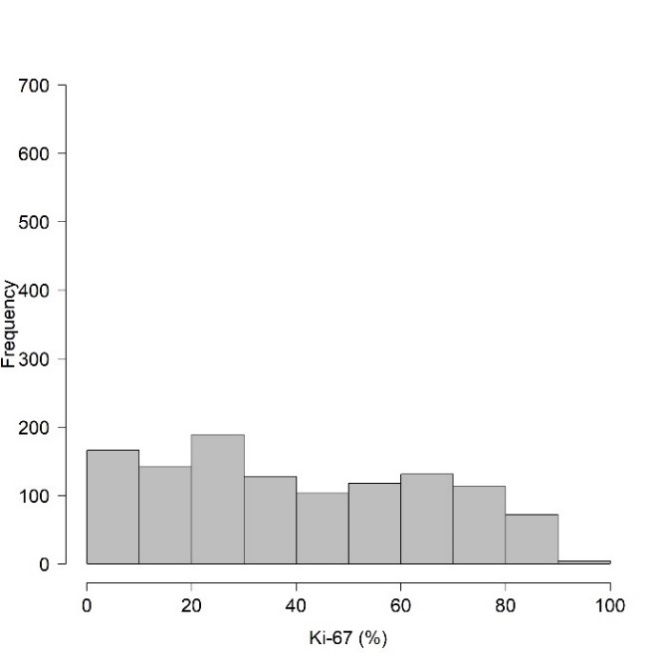** |  |

Supplementary Figure S2**: Predicted probability of pCR as a continuous function of ER, PgR, and Ki-67 (a) without considering further predictors and (b) with predictions obtained from the final multivariate prediction model.** The curves in (**b**) show the pCR likelihood for a hypothetical average patient—ie, a patient of median age (52 years) with median body mass index (25.1 kg/m^2^) and belonging to the most frequent categories (cT2, G3, cN+, ductal). Solid curves predict pCR likelihoods for an average patient with average biomarker expression in two out of three predictors (median ER, 60%; median PgR, 4%; median Ki-67, 40%). The curves with long dashes predict pCR likelihoods for an average patient with increased pCR probability due to biomarker expressions (ER, 10th percentile, 0%; PgR, 10th percentile, 0%; Ki-67, 90th percentile, 80%). The curves with short dashes predict pCR likelihoods for an average patient with decreased pCR probability due to biomarker expressions (ER, 90th percentile, 90%; PgR, 90th percentile, 83%; Ki-67, 10th percentile, 10%). [ER=estrogen receptor; pCR=pathological complete response; PgR=progesterone receptor]

| **a**  **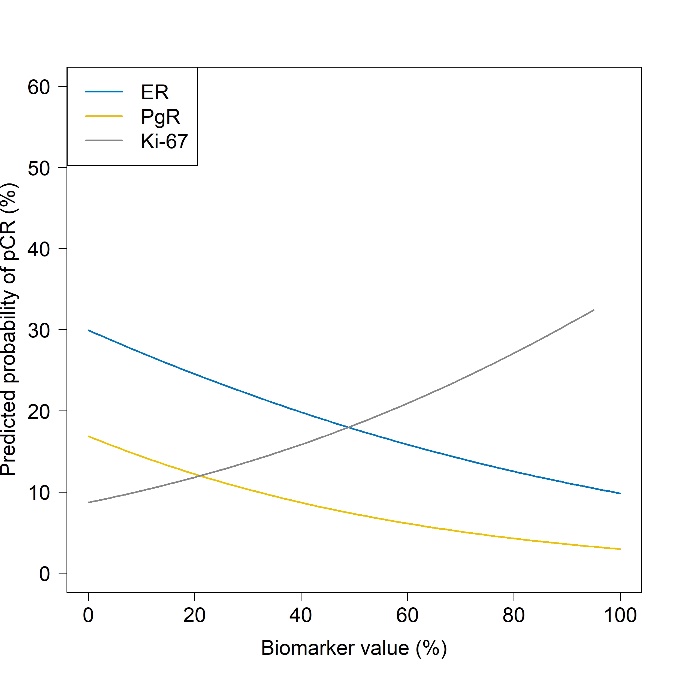** | **b**  **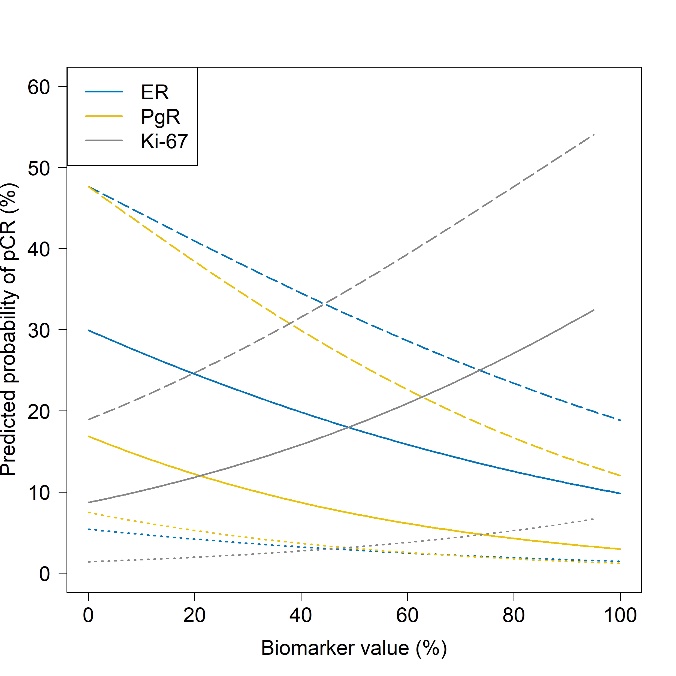** |
| --- | --- |

Supplementary Figure S3:**Cutoff point calculations for predicting a pathological complete response, showing the deviances of simple logistic regression models with the binary predictor (at or below cutoff point vs above cutoff point): (a) estrogen receptor, (b) progesterone receptor, and (c) Ki-67 as a function of cutoff points.** The smaller the deviance, the better the separation of the patient groups. The deviance is in 1:1 correspondence to the *P* value of the global likelihood ratio test.

| **a**  **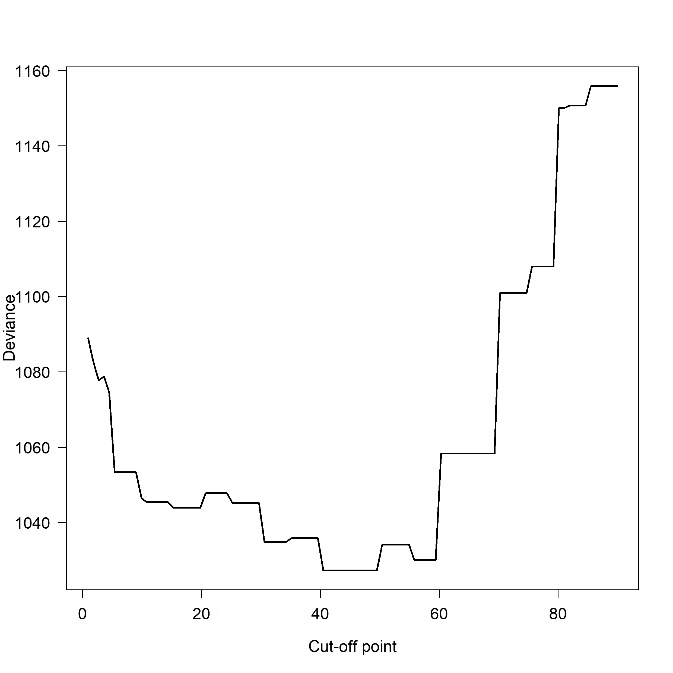** | **b**  **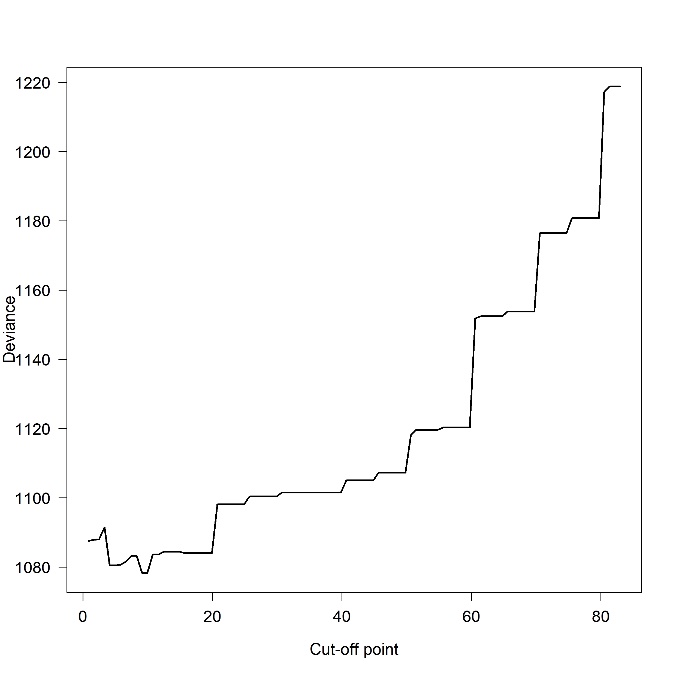** |
| --- | --- |
| **c**  **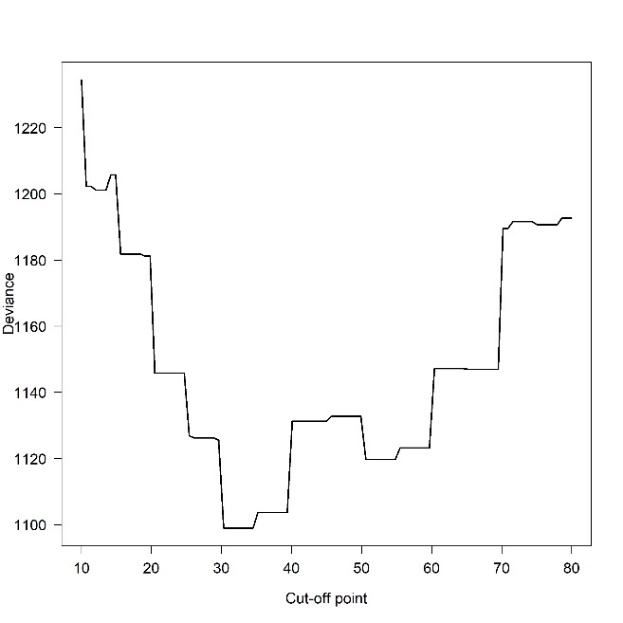** |  |

Supplementary Figure S4: **Distributions of the predicted pCR likelihoods (pCR-prob, 0%**–**100%) in the primary study population.** The bars show how many patients had a predicted likelihood between 0% and 5%, between 5% and 10%, etc. Half of all patients had a predicted likelihood between 3.4% and 43.2% (IQR). The median was 14.1%. Distributions are shown for (**a**) all patients, (**b**) patients with triple-negative disease (median, 45.7%; IQR, 34.3%–56.5%), and hormone receptor–positive patients with (**c**) tumor grade 1 or 2 (luminal A–like; median, 2.6%; IQR, 1.5%–5.5%), and (**d**) tumor grade 3 (luminal B–like; median, 12.7%, IQR, 5.7%–27.8%) [IQR=interquartile range; pCR=pathological complete response; pCR-prob=probability of pathological complete response]

| **a**  **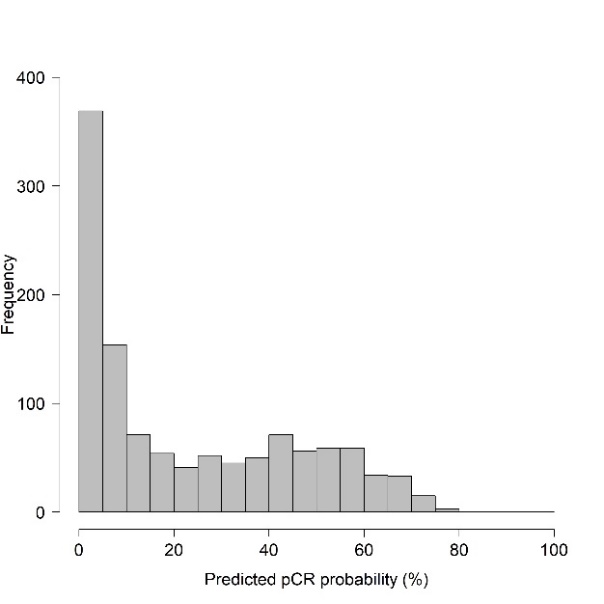** | **b**  **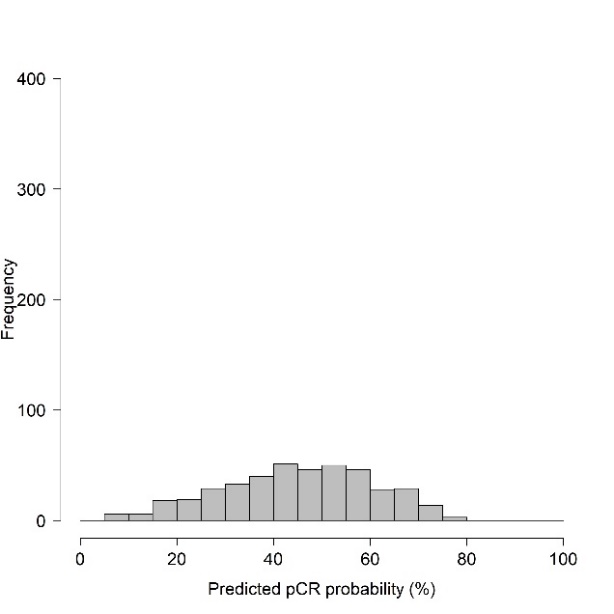** |
| --- | --- |
| **c**  **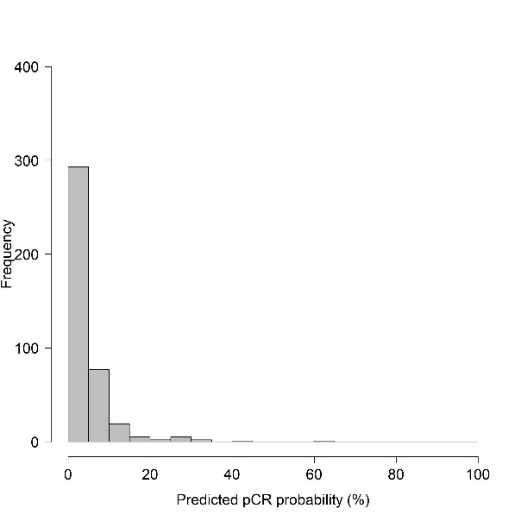** | **d**  **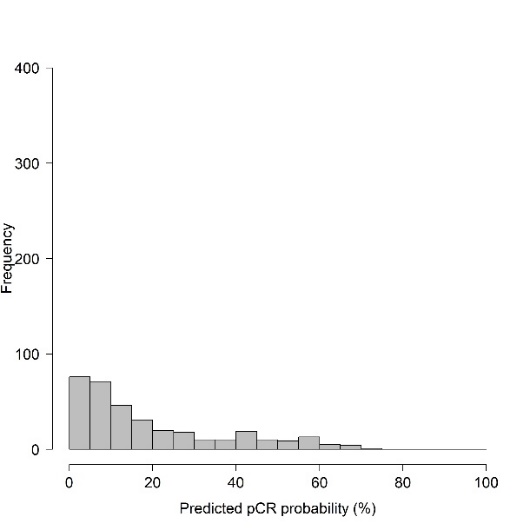** |

Supplementary Figure S5: **The observed and predicted frequencies of pCR.** (**a**) Primary study cohort. Validation cohorts: (**b**) HaBCS, (**c**) GeparSepto paclitaxel arm, (**d**) GeparSepto nab-paclitaxel arm, (**e**) GeparOcto ETC arm. The patients were sorted relative to the predicted probability of pCR using the linear biomarker prediction model and grouped into categories based on percentiles. The number of actually observed pCRs (termed observed events) in each category and the sum of predicted probabilities for pCR (termed predicted events) in each category are shown. Filled circles are used for predictions from the original prediction model, and triangles are used for predictions from a recalibrated prediction model (**c**, **d**). Points below the diagonal indicate when the model is overestimating the likelihood of pCR; points above the diagonal indicate when the model is underestimating the likelihood. A perfect prediction model would show all points on the diagonal. [ETC=high-dose epirubicin, taxane, and cyclophosphamide; HaBCS=Hannover Breast Cancer Study; pCR=pathological complete response]

| **a – primary study cohort HaBCS**  **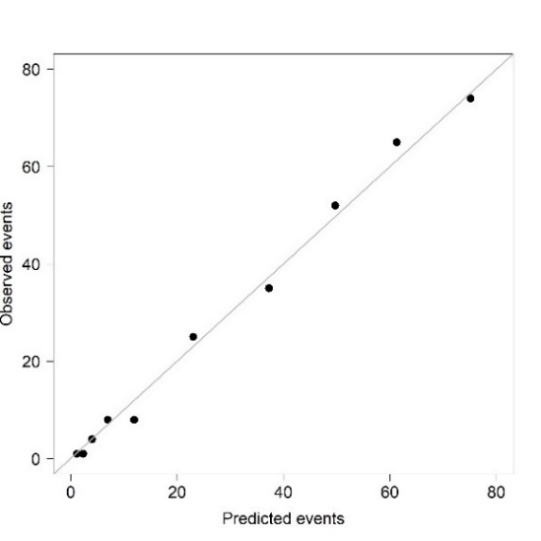** | **b – HaBCS**  **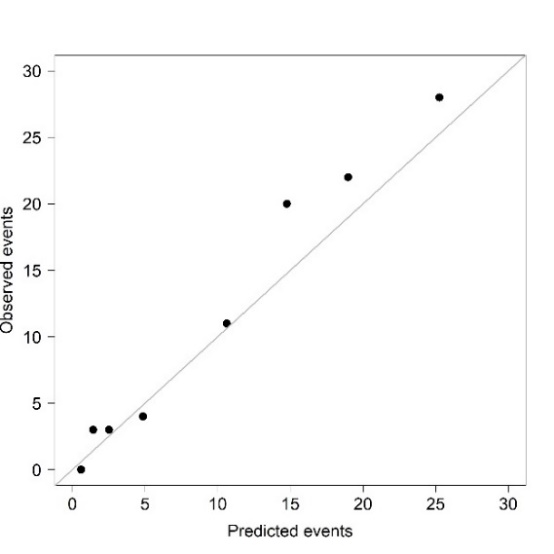** |
| --- | --- |
| **c – GeparSepto – paclitaxel arm**  **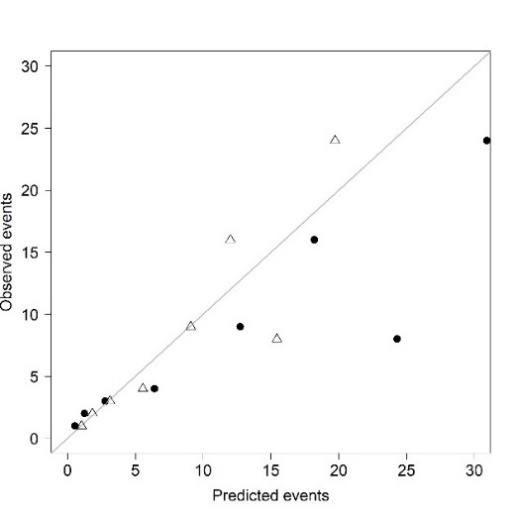** | **d – GeparSepto nab-paclitaxel arm**  **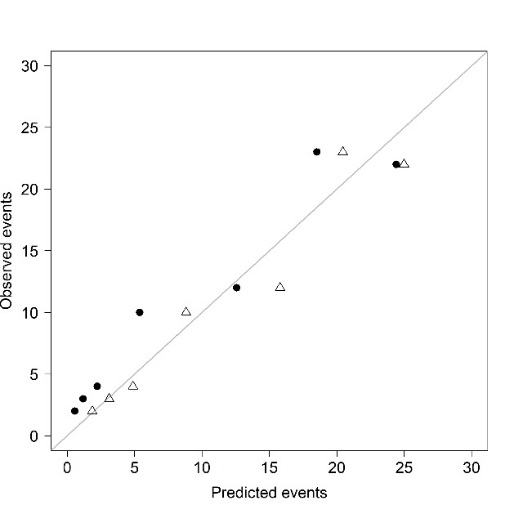** |
| **e – GeparOcto – ETC arm**  **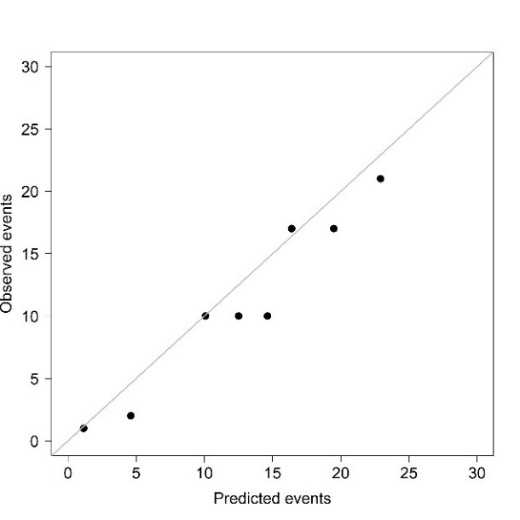** |  |

Supplementary Figure S6**:  ROC curves for the final prediction model of pCR in the primary study cohort (showing cross-validated results) and in external validation cohorts.** [ETC=high-dose epirubicin, taxane, and cyclophosphamide; HaBCS=Hannover Breast Cancer Study; pCR=pathological complete response; ROC=area under the receiver operating characteristic curve]

**
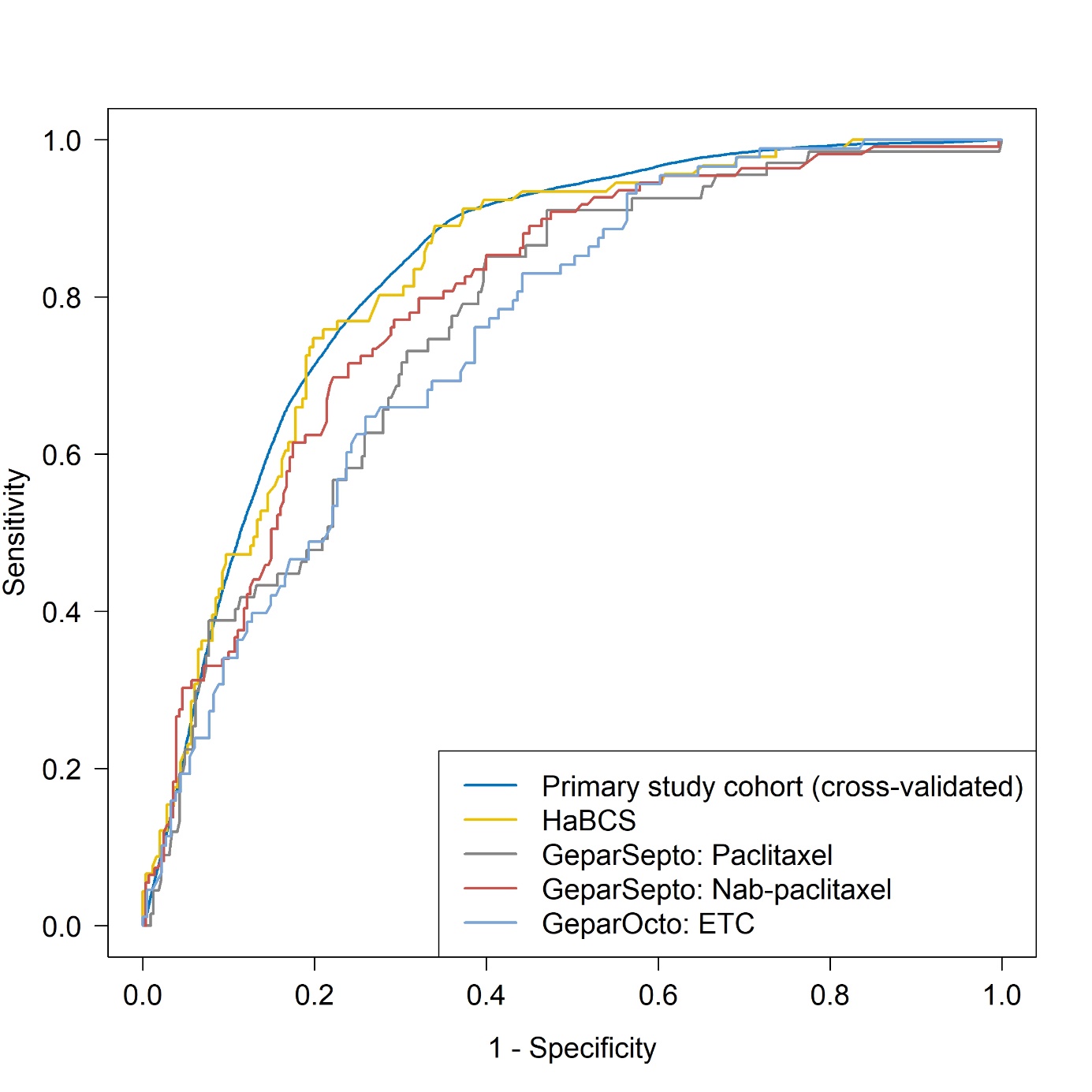
**

Supplementary Figure S7: **Comparison of pCR probabilities with IHC3+C recurrence risk scores in the primary study population.** The gray vertical and horizontal bars indicate the 25th, 50th, and 75th percentiles of the distribution. [IHC3+C=estrogen receptor, progesterone receptor, Ki-67, and clinical predictors; pCR=pathological compete response]

**
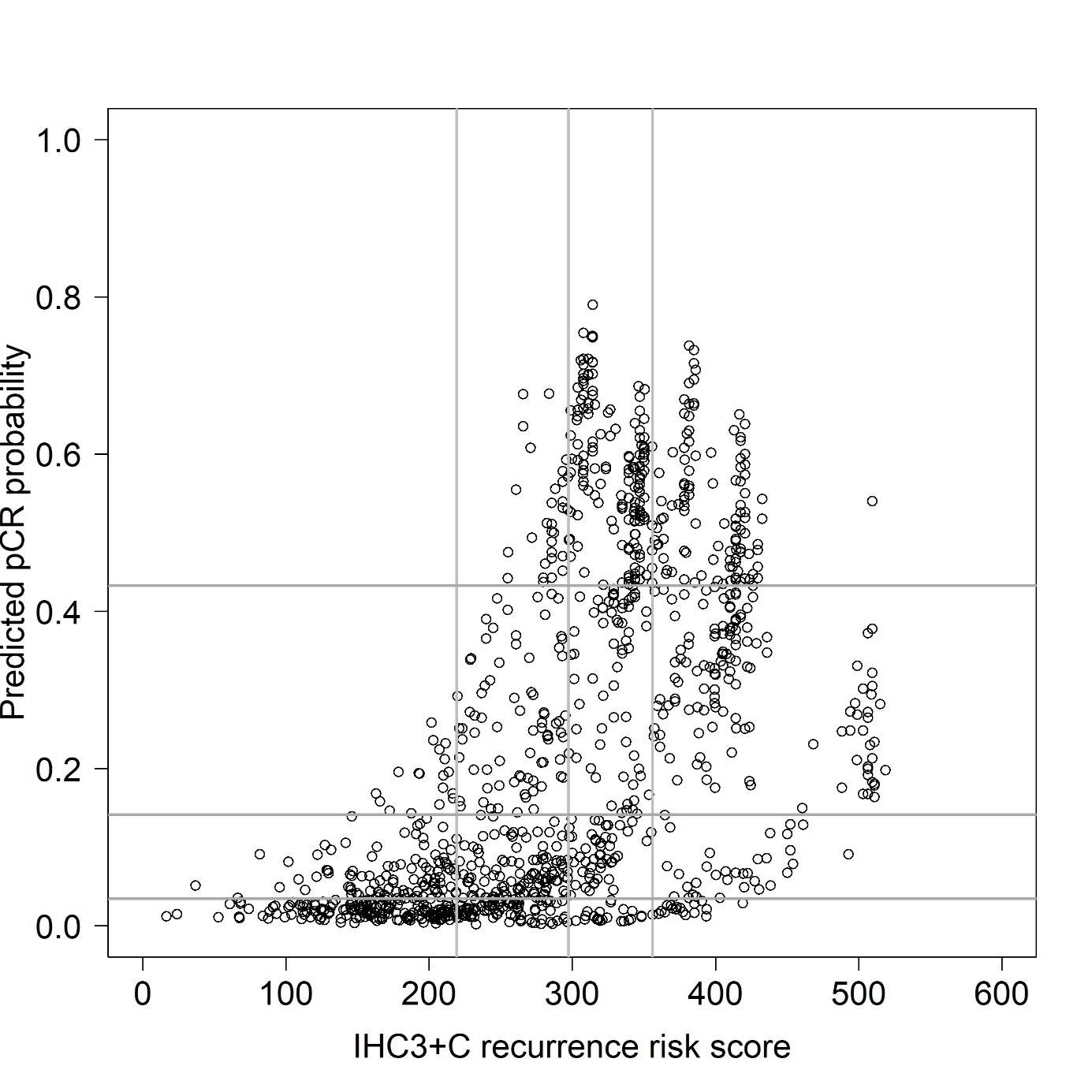
**

Supplementary Figure S8: **Kaplan-Meier estimates for disease-free survival relative to IHC3+C classes** (low, <210, first quartile; intermediate, 210-350, interquartile range; high ≥350, third quartile) [IHC3+C=estrogen receptor, progesterone receptor, Ki-67, and clinical predictors]


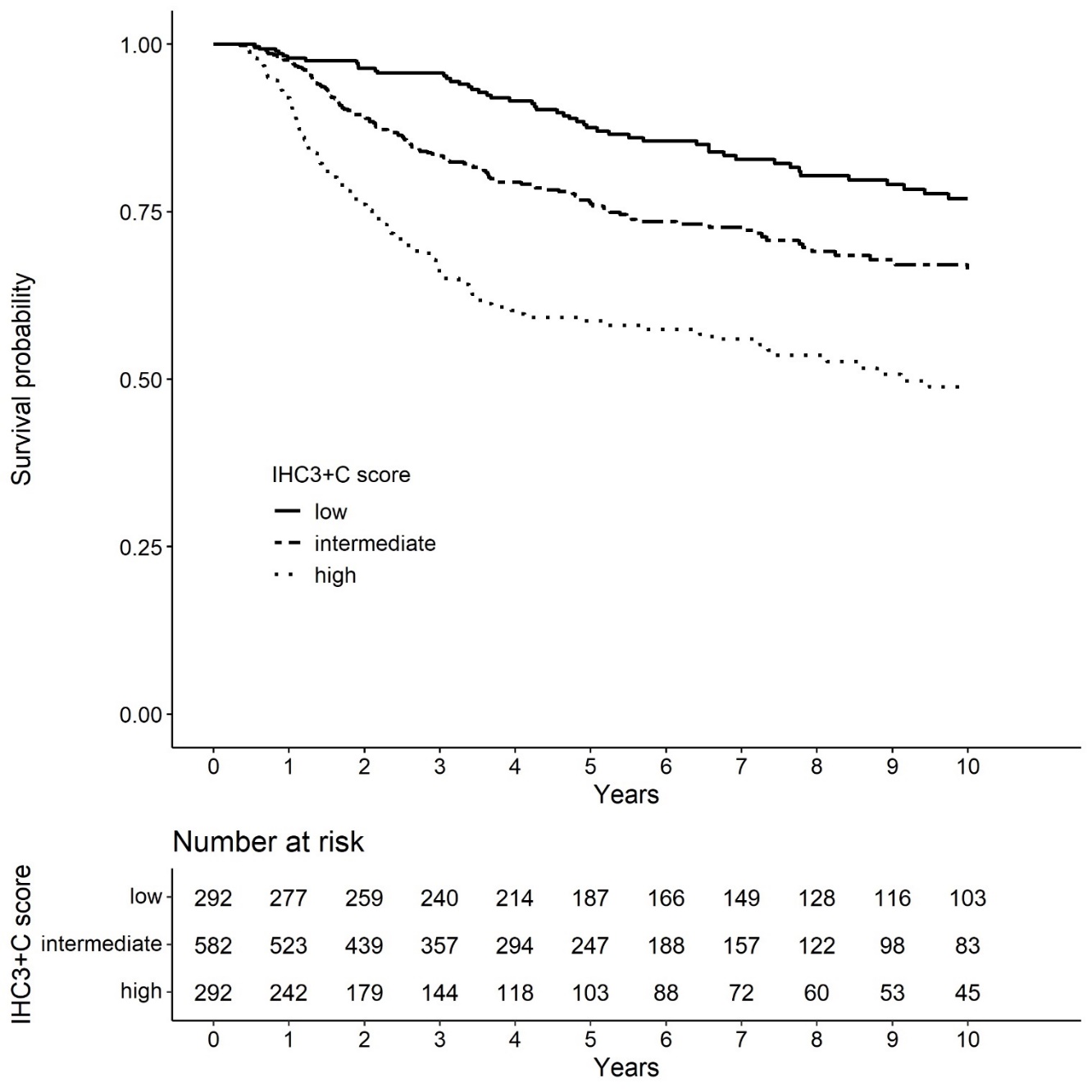

Supplement: Supplementary file 1 — Additional file 1. [file 13058_2025_1960_MOESM1_ESM.docx]
